# Hydrogen Separation Membranes: A Material Perspective

**DOI:** 10.3390/molecules29194676

**Published:** 2024-10-01

**Authors:** Dixit V. Bhalani, Bogyu Lim

**Affiliations:** Department of Engineering Chemistry, Chungbuk National University (CBNU), Cheongju 28644, Chungbuk, Republic of Korea

**Keywords:** hydrogen purification, polymer membranes, sustainable energy, mixed-matrix membranes, membrane materials

## Abstract

The global energy market is shifting toward renewable, sustainable, and low-carbon hydrogen energy due to global environmental issues, such as rising carbon dioxide emissions, climate change, and global warming. Currently, a majority of hydrogen demands are achieved by steam methane reforming and other conventional processes, which, again, are very carbon-intensive methods, and the hydrogen produced by them needs to be purified prior to their application. Hence, researchers are continuously endeavoring to develop sustainable and efficient methods for hydrogen generation and purification. Membrane-based gas-separation technologies were proven to be more efficient than conventional technologies. This review explores the transition from conventional separation techniques, such as pressure swing adsorption and cryogenic distillation, to advanced membrane-based technologies with high selectivity and efficiency for hydrogen purification. Major emphasis is placed on various membrane materials and their corresponding membrane performance. First, we discuss various metal membranes, including dense, alloyed, and amorphous metal membranes, which exhibit high hydrogen solubility and selectivity. Further, various inorganic membranes, such as zeolites, silica, and CMSMs, are also discussed. Major emphasis is placed on the development of polymeric materials and membranes for the selective separation of hydrogen from CH_4_, CO_2_, and N_2_. In addition, cutting-edge mixed-matrix membranes are also delineated, which involve the incorporation of inorganic fillers to improve performance. This review provides a comprehensive overview of advancements in gas-separation membranes and membrane materials in terms of hydrogen selectivity, permeability, and durability in practical applications. By analyzing various conventional and advanced technologies, this review provides a comprehensive material perspective on hydrogen separation membranes, thereby endorsing hydrogen energy for a sustainable future.

## 1. Introduction

The conventional fossil fuel-based economy has raised several issues, among which climate change, global warming, and increasing CO_2_ emissions are the focal point of discussion these days. From the start of the industrial revolution until now, CO₂ emissions have exponentially increased [1,2,3]. Additionally, the growing population, fossil fuel consumption, reliance on conventional energy sources, urbanization, and rapid industrialization have led to the excessive addition of greenhouse gases into the atmosphere [4,5,6]. Fossil fuel reserves are also rapidly depleting [7,8,9]. Hence, to reduce fossil dependency and protect the ecosystem, research has focused on the development of sustainable, renewable, and clean energy sources capable of meeting future global energy demands and helping to achieve the climate goal of limiting the global temperature rise of 1.5 °C [10,11]. Hydrogen has emerged as a potential alternative for sustainable future energy developments in the post-fossil fuel period [12,13]. It is capable of mitigating the global energy crisis and global warming issue simultaneously, ensuring future energy security [14,15]. Hydrogen can be employed for a broad range of applications, such as in industrial feedstock, electricity generation, energy storage/carriers, and transportation (fuel). It is a zero-emission fuel, generating only water vapor as a byproduct at the end of combustion. These qualities make it a potential sustainable alternative to non-renewable fossil fuels, which generate a tremendous amount of greenhouse gases and cause significant harm to the environment.

As it can be produced domestically, hydrogen is considered one of the most promising energy security options globally. It reduces the dependency on imported energy (petroleum and other fossil fuels) and creates domestic employment in the field of energy generation [15]. The energy crisis occurred due to COVID-19, and the Russian invasion in Ukraine has further intensified the debate on energy dependency on fossil fuels and imported foreign fuels in European governments [16,17]. As the utilization of hydrogen fuel is currently in the developmental stage, its transition to an alternative sustainable energy source will take a long time. Continuous monitoring and promotive steps by governments will accelerate its development and transition. Hassan discussed the policies and strategies implemented by the USA, Japan, Australia, Canada, and the European Union to expedite developments and for the utilization of green hydrogen technologies. Various challenges and limitations on the path of green hydrogen were also discussed, such as infrastructures, technical issues, policies, economic feasibility, and public acceptance and perceptions [18].

Unlike conventional energy sources, such as natural gas, coal, and petroleum, hydrogen is not the primary form of energy. It needs to be generated using other energy sources, either renewable or non-renewable, i.e., nuclear, natural gas, coal, biomass, wind, photovoltaic energy, and geothermal energy. This wide range of sources makes hydrogen a highly promising energy alternative for future energy security [19,20,21,22]. For the substitution of current fossil fuel-based energy systems with hydrogen-based ones, an immense amount of hydrogen will need to be generated.

**Figure 1:**
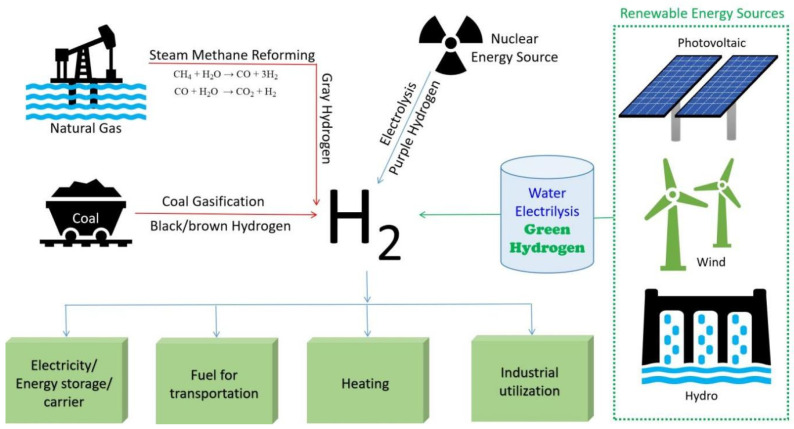
Schematic representation of various raw material sources and energy sources for hydrogen production and its diversified applications.

Researchers are continuously working on the development of hydrogen generation from non-conventional and renewable energy sources. Currently, steam methane reforming (SMR), biomass and coal gasification, and water electrolysis are the major industrial processes used (Figure 1). Among these, the economic viability of SMR and gasification make them suitable for industrial-scale H_2_ generation. SMR involves the reaction of methane with steam in the presence of a nickel catalyst at 700–1100 °C (reforming reaction), which generates a mixture of hydrogen and carbon monoxide (jointly called syngas). Furthermore, the reaction of CO with steam through the water–gas shift reaction generates additional H_2_ [23,24,25]. In the gasification technique, syngas has been produced by a reaction of steam with municipal solid waste, coal, or biomass, which further produces H_2_ through the water–shift gas reaction [26,27]. The generated gas mainly contains H_2_ and CO_2_, with the presence of a trace amount of contaminants, for example, H_2_S, CH_4_, and CO. However, the application of H_2_ in fuel cells requires high purity H_2_ (~99.99%); hence, prior to application, the H_2_ stream needs to be purified to eliminate water vapors, CO, CO_2_, H_2_S, CH_4_, C_2_H_6_, chlorides, NH_3_, and trace amounts of other catalyst-poisoning impurities [19]. The electrolysis process, generally known as water splitting, involves the breaking of water into H_2_ and O_2_ by means of electricity. Based on the electrolyte used, it may be further categorized as proton exchange membrane water electrolysis (PEME), alkaline water electrolysis (AWE), anion exchange membrane water electrolysis (AEME), or solid oxide water electrolysis (SOEC) [28,29]. Guan et al. discussed the techno-economic assessment and commercial feasibility of these water electrolysis technologies. AWE, PEME, and AEME involve low-temperature water splitting (<90 °C), and SOEC involves high-temperature water splitting (>300 °C). Among these, PEME and AWE are commercially feasible technologies, while AEME and SOEC are comparatively immature and remain at the laboratorial stage. As AWE does not involve the use of precious metals, it is more cost-effective than PEME, which involves the use of noble metals. By techno-economic assessment, these authors revealed that the hydrogen production cost follows the order of H_2_ from coal and natural gas (gray hydrogen) < AWE < PEME < SOEC (waste heat) < SOEC. The cost was calculated considering operational costs, capital costs, maintenance, feedstocks, and replacements. Here, electricity consumption (feedstocks) contributed to most of the costs, namely, AWE contributed ~72.9%, PEME contributed ~64.0%, SOEC contributed ~45.2%, and SOEC with waste heat contributed ~36.7% with respect to the total cost. The hydrogen produced from these technologies needs to be purified prior to its application in fuel cells, as they have some impurities, mainly, water vapor and oxygen. The H_2_ produced by AWE contains water vapor, oxygen, and trace amounts of KOH as an impurity, which can be detrimental to the fuel cell catalyst of proton exchange membrane fuel cells (PEMFCs). The H_2_ produced by PEME and SOEC is of high purity but still contains trace amounts of water vapor and oxygen, which need to be eliminated before their application in fuel cells. The H_2_ produced from these green hydrogen technologies (AWE, PEME, and SOEC) is of a comparatively lower amount and contains less toxic impurities, but it still needs to be integrated with membrane separation technology [30].

Compared to SMR and gasification, water electrolysis is a very environmentally friendly method for H_2_ generation, but due to its high cost, its contribution to the global H_2_ generation is still limited to ~4% [31,32]. On the other hand, SMR is a low-cost and economically viable technology for large-scale H_2_ generation in industries. SMR accounts for more than 80% of the global H_2_ generation. The economic limitations of other renewable sources based H_2_-generation methods make fossil fuel-based H_2_ a practical option in this transitional period of a fossil fuel-based economy to a hydrogen economy. Hence, in the early stages of a hydrogen economy, it is expected that fossil fuel-based H_2_ generation will be integrated with CO_2_ sequestration [33,34].

As discussed above, hydrogen produced from SMR and gasification needs to be purified from residual gases, i.e., CO_2_, CH_4_, and other impurities. Fuel cells and other industrial applications require high-purity hydrogen, making H₂ purification critically important in order to achieve a hydrogen economy. The platinum-based catalysts in PEM fuel cells are very sensitive to H_2_S and CO poisoning, which has led to a focus on the development of more efficient H_2_ purification methods [19,35,36].

Pressure swing adsorption (PSA) technology, cryogenic distillation, and amine-based absorption are the most widely employed techniques for H_2_ purification [37,38]. PSA uses several beds of solid adsorbents, which are capable of selectively adsorbing impurities (such as water vapor, CO, and CO_2_), producing 99.99% of pure H_2_. Based on their affinity, the impurities are physically bonded with the solid adsorbent. Hydrogen, being of a highly volatile nature and having a low polarity, cannot be adsorbed by the beds, while N_2_, water vapor, CO, CO_2_, and other impurities are readily adsorbed by the beds. The separation efficiency depends on the characteristics of the impurities (such as their partial pressure), the type of adsorbent, and the binding forces involved. PSA works on alternative cycles of adsorption and desorption at a constant temperature. These cycles are performed in very short time spans, allowing for the removal of a large amount of impurities at a low cost. Firstly, adsorption is carried out at a high pressure (10–40 bar) until equilibrium is achieved. Then, adsorption beds are further regenerated by reducing the pressure, which causes the desorption of impurities from the beds. The regenerated beds are again pressurized to adsorb the impurities [37,38,39]. This cyclic process of adsorption and desorption is much more economical but has limitations in recovery, with a loss of around ~20% of H_2_. PSA technology is generally employed in integrations with steam methane reforming units to produce fuel cell-quality H_2_ [19].

Cryogenic distillation involves separation by the partial condensation of gas mixtures at a high pressure and a low temperature. This process is non-economical due to the requirement of expensive devices and equipment. It is also not competitive in terms of purity, with a limited efficiency of up to ~99% [37,40]. Cryogenic distillation and PSA are both processes that need high-cost equipment facilities and are associated with a greater energy uptake. H_2_ production by SMR technology and gasification is economical, but the purification step accounts for ~50% of the cost. Still, the low production cost makes SMR and gasification more economical compared to electrolysis. Further developments in separation technologies will make them a more viable option in the era of transition from a non-renewable to a renewable energy economy [19,37]. Existing hydrogen plants are associated with industries and are dedicated to the industrial application (feedstocks) of hydrogen. However, hydrogen facilities for the energy sector (storage/carriers); fuel generation (in the transportation sector, including shipping, aviation, and vehicles); and heat generation are currently in the developmental stage. Hosseini provided a detailed study on the current and projected future demand for hydrogen in the transportation sector [15]. Razi and Dincer discussed the challenges and scope of hydrogen utilization in the aforementioned sectors in Canada [41]. Hence, an economically viable technique with high separation efficiency is required.

To this end, H_2_-selective membrane separation technology emerged as a promising alternative to conventional separation techniques [42]. The membrane is a thin selective barrier that allows for the selective component to permeate. Compared to other techniques, membrane technology offers high energy efficiency, operating versatility, a compact design, low carbon footprints, a low operational cost, the ease of scale-ups, and the ease of integration with active industrial processes [43]. The gas-separation membrane is driven by either a difference in the partial pressure or electrical potential.

Various types of membranes have been developed and used for H_2_ purification, such as metallic membranes; inorganic membranes (carbon molecular sieves (CMSMs), zeolite, and silica membranes); and polymeric membranes [44,45]. However, polymeric membranes are limited by a trade-off between selectivity and permeability, known as the Robeson upper-bound limit [46]. Therefore, they need to be improved in terms of selectivity and permeability, which can be achieved by developing new membrane materials and approaches. Inorganic materials, such as silica, zeolites, and various metallic membranes, have already been proven to be promising materials for hydrogen purification [47]. Pd, Pt, and other metals of groups IV–V have a great potential for their use as hydrogen separation membranes. Palladium-based membranes offer superior separation efficiency over a broad temperature range. However, they are of a high cost and are difficult to process on a large scale [48]. Over time, various polymeric membranes have been developed to overcome the limitations of conventional membranes. Various inorganic materials have been blended into the polymer phase to fabricate hybrid membranes, termed as mixed-matrix membranes (MMMs) [45,49]. The exponential growth of the hydrogen economy has fueled advancements in membranes, membrane materials, and membrane-based separation technologies for H_2_ purification. Currently, novel advanced materials such as functionalized polymers, MOFs, thermally rearranged polymers, and grapheme-based materials are also being employed for the fabrication of H_2_-selective membranes [50,51].

In the present review, we present various membrane technologies for H_2_ purification, with a strong emphasis on H_2_-selective polymeric membranes and mixed-matrix membranes. First, we discuss the preparation, uses, and limitations of various conventional membranes, including metallic membranes and inorganic membranes (zeolite, silica, and carbon molecular sieve membranes). Further, polymeric membranes are discussed in three separate sections covering the following types: H_2_/CO_2_, H_2_/CH_4_, and H_2_/N_2_ separation membranes. Next, recent advancements in mixed-matrix membranes are elaborated on. This review provides an overview of the various materials used in hydrogen purification membranes, including inorganic, organic (polymeric), and mixed blends. We anticipate that this brief overview of membrane materials and H_2_-selective membranes will be helpful for further advancements in membrane materials and membrane technologies for H_2_ enrichment, thereby supporting the future of energy.

## 2. Conventional Membranes

### 2.1. Metal Membranes

Dense metallic membranes have higher thermal stability and H_2_ selectivity, which makes them crucial for H_2_ separation from raw mix gaseous streams. They are generally used in the case of chemical synthesis and fuel cells, where high-quality H_2_ is required [41]. The basic criteria for this membrane are to restrict the impurities and selectively pass hydrogen. Here, the dense metallic structure restricts CO_2_, N_2_, and CH_4_, which are larger gas molecules, and provides the selective permeation of H_2_ through the “solution–diffusion mechanism”. This process involves several steps: (i) the adsorption of H_2_ onto the membrane surface, (ii) the dissociation of H_2_ into atomic hydrogen, (iii) the dissolution of atomic H in the bulk membrane, (iv) the diffusion of hydrogen through the metal lattice, (v) the reassociation of H atoms to the H_2_ molecule, and (vi) the desorption of pure H_2_ from the membrane surface [52,53].

**Figure 2:**
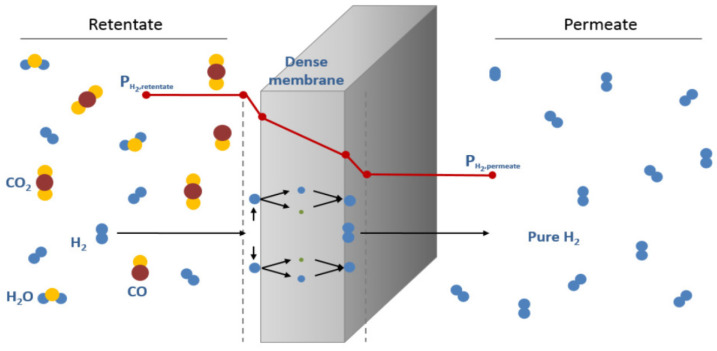
Schematic representation for hydrogen purification by dense metallic membranes through the solution–diffusion mechanism. Reprinted from ref. [55].

Initially, the H_2_ molecule undergoes physisorption, further binding to the active sites of the metallic surface through chemical bonding. In dissociation, the chemisorption of H_2_ molecules with the metal surface weakens the H–H bond, which gets further broken down to form two individual H atoms. The dissociated H atoms travel across the metal membrane through diffusion, which further recombine and desorb from the metal surface as H_2_ molecules (Figure 2). The dense structure of the metal membrane possesses high selectivity, resulting in a high-purity product stream. Compared to other materials, the thermal stability of metallic membranes offers operability at elevated temperatures [45,47,54,55]. Here, during the separation process, the partial pressure of H_2_ on the feed side (retentate side) of the membrane is called PH_2_ retentate, and the partial pressure of hydrogen on the permeate side of the membrane, where the pure hydrogen emerges after passing through the membrane, is called PH_2_ permeate. As the difference between the PH_2_ retentate and the PH_2_ permeate creates the driving force for hydrogen permeation through the membrane, a higher pressure on the retentate side and a lower pressure on the permeate side typically enhance the rate of hydrogen permeation.

Among all metallic membranes, Pd membranes have been the most extensively explored due to their greater hydrogen solubility and high selectivity [56,57,58,59]. Pd membranes have a high tolerance toward hydrogen embrittlement and have a superior catalytic ability for hydrogen dissociation and recombination compared to other metal membranes [60]. However, the high sensitivity of Pt and Pd membranes toward contaminants such as thiophene, iodine, H_2_S, and CO drastically reduces their performance [61,62]. The development of defect-free thin-layered Pd membranes with high chemical stability, thermal stability, and durability is still a challenge. Hydrogen diffusion is higher in a BCC (body-centered cubic) (group V elements: V, Nb, and Ta) than an FCC (face-centered cubic) (group X elements: Pd, Pt, etc.) lattice. Also, Pd-based membranes are very expensive compared to niobium-, vanadium-, and tantalum-based membranes. These metals, from group V (with a BCC structure), possess higher H_2_ permeability compared to Pd-based membranes [58,63]. However, group V metals are prone to oxidation, which further affects their transport properties. The hydrogen dissociation ability of these metals is low as compared to Pd; hence, an additional catalytic layer is needed. Meanwhile, Pd, with its self-catalyzing ability, does not require any additional layer. Also, Pd is a noble metal with a high resistance to oxidation and high hydrogen solubility at room temperature. However, a pure Pd membrane is still somewhat prone to structural deformation due to lattice expansion from hydrogen absorption, which can cause cracks and pinholes [48,63]. And the Pd membrane catalytic surface is easily poisoned by impurities such as CO, H_2_S, Hg, NH_3_, and CO_2_. H_2_S causes the formation of a low-permeable Pd_4_S layer on the Pd membrane surface, consequently decreasing the H_2_ permeability [64,65].

To counter the issues of hydrogen embrittlement, hydride formation, contaminant poisoning, and low mechanical strength, metal alloys were developed to improve performance, stability, and strength and to reduce the cost of membranes. They are generally categorized as Pd-based alloys and non-Pd alloys. Palladium is generally alloyed with Ce, Ni, Pt, Ru, Y, Au, Cu, or Ag. Metals from groups IV and V are alloyed with Mn, Ni, Ge, Si, Fe, Mo, Ga, Cu, Sn, La, Be, or W to improve the degradation resistance, strength, and durability [54,63,66,67]. Small amounts of Mo, Rh, Zr, and Ru can minimize the metal-membrane embrittlement caused by hydride formation. Metal alloying with Ag, Cu, Fe, or Ni protects against toxic impurities, such as water vapor, H_2_S, and CO [54]. Several binary and ternary alloys have been prepared and investigated for their improved properties and performance in hydrogen purification. Other strategies, such as coating with protective materials against poisoning, surface functionalization, and catalytic coating, have also been investigated to improve performance and durability [68,69].

**Figure 3:**
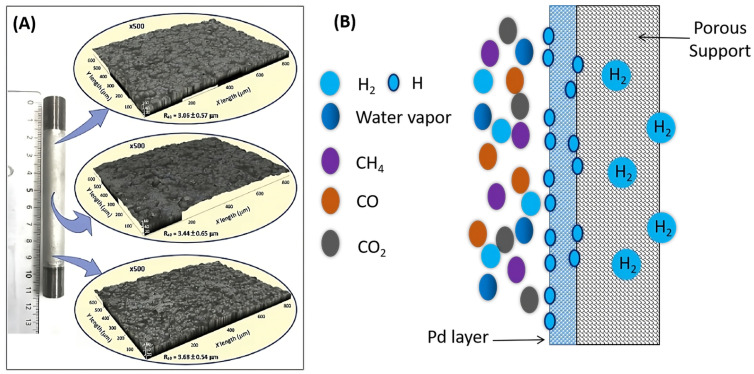
(**A**) Digital image of stainless-steel-supported palladium membrane prepared by ELP–PP (electroless plating–pore plating) and its subfigures shows the external surface roughness measured at different areas. The measured roughness values are 3.06 ± 0.57 (**upper**), 3.44 ± 0.65 (**middle**) and 3.68 ± 0.54 μm (**lower**). Reprinted with permission from ref. [60]. (**B**) Schematic representation of supported Pd membrane.

Supported Pd-based membranes were developed to improve the H_2_ flux, minimize the thickness of the selective layer, and reduce the cost of materials. Supports made from stainless steel [60], ceramics, Vycor glass, and porous nickel were used to prepare supported Pd-based membranes (Figure 3). Weber et al. presented a Pd-/Al_2_O_3_-based hydrogen-selective composite membrane (supported Pd-based membranes). The Pd nanoclusters were immobilized in porous γ-Al_2_O_3_ by atomic layer deposition. The membranes were reported to have a higher hydrogen permeation rate, above 1000 GPU, and a separation factor of (H_2_/CO_2_) ~9 and (H_2_/N_2_) ~16 [43,55,70,71].

Several chemical and physical methods have been reported in the literature to prepare Pd membranes with the desired thickness. The chemical methods include chemical vapor deposition (CVD), electroless plating deposition (ELP), electrochemical vapor deposition (EVD), the sol–gel technique, molecular layering (ML), electroplating (EP), solvated metal atom deposition, and spray pyrolysis. The physical methods include sputtering, magnetron sputtering (MS), physical vapor deposition (PVD), and conventional cold rolling [47,55].

Amorphous metal membranes possess greater structural and mechanical properties compared to their crystalline-equivalent metal membranes. They offer higher ductility, strength, corrosion resistance, and H_2_ permeability than their crystalline equivalents [72]. An amorphous membrane having a more open lattice reduces the risks associated with hydrogen embrittlement [73,74]. It can sustain an elevated temperature and pressure for a greater number of cycles, which makes it most suitable for industrial-scale H_2_ purification. The amorphous membrane has higher flexibility and enhanced catalytic-surface activity, which facilitates higher surface–hydrogen interactions [75]. Several reviews have summarized various metallic membranes, including Pd-based membranes, alloyed ones, and supported membranes, along with their operating conditions and corresponding permeability and selectivity [54,71,76]. In the present review, similar data are summarized in Table S1 of the supporting information.

Their high selectivity, thermal stability, chemical resistance, and high-temperature operability enable metal membranes, especially Pd membranes, to be used in commercial applications. However, the scaling-up of metal membranes is still challenging due to the high cost of precious metals, complex manufacturing methods, and durability issues. They are also susceptible to poisoning by CO and sulfur. To overcome the issues of durability and metal costs, alloyed membranes and thin-film-supported membranes have been developed. Hence, costs can be reduced while maintaining selectivity and performance.

### 2.2. Zeolite Membranes

Zeolites are a three-dimensional (3D) crystalline network of aluminosilicate with a uniform pore structure, which are employed for various applications, including ion exchanges, catalysis, adsorption, and membrane-based separations. Their high chemical resistance, thermal stability, mechanical stability, adjustable pore size, and molecular sieving ability make them suitable for H_2_ purification. Different zeolite frameworks, such as MFI, DDR, LTA, CHA, and FAU, have been used to prepare membranes. Their structure-specific properties make them suitable for the various applications mentioned above. Self-standing zeolite membranes cannot sustain themselves, due to their brittleness, and are also challenging to produce on a large scale; hence, tubular or disk-shaped porous supports made from alumina (α-Al_2_O_3_), ZrO_2_, stainless steel, TiO_2_, and polymer supports are used [45,77,78,79]. This support improves gas permeability and mechanical strength [80].

Zeolite membranes are generally prepared by the in situ crystallization method (hydrothermal), secondary growth (hydrothermal), pore plugging, steam-assisted crystallization, and vapor-phase transport [81,82,83,84]. Recently, the ionothermal method has gained much attention. The ionic liquid works as a reaction medium and also acts as a structure-directing agent. Hence, by using different ionic liquids, the structure and properties of zeolite can be tuned. Li et al. prepared AlPO-based membranes on an alumina support using the ionothermal technique [85].

**Figure 4:**
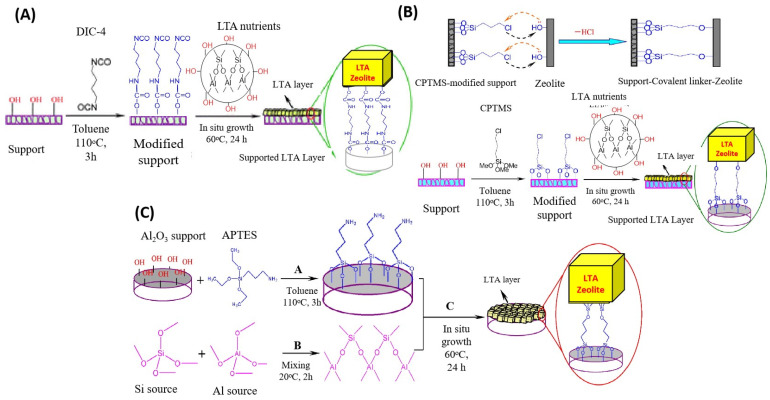
Schematic representation for the fabrication of zeolite LTA membrane on a functionalized support using (**A**) 1,4-diisocyanate (DIC-4) as the covalent linker, (**B**) 3-chloropropyltrimethoxysilane (CPTMS) as the covalent linker, and (**C**) 3-aminopropyltriethoxysilane (APTES) as the covalent linker. Reprinted with permission from refs. [88,89,90].

Various LTA, DDR, MFI, and SAPO-34 membranes have been investigated for H_2_/CO_2_ separation [45,86,87]. However, the inner crystalline defects range from 1 to 2 nm and are comparatively much bigger than H_2_ and CO_2_ (0.289 and 0.330 nm), compromising the membrane’s performance. Hence, various strategies have been employed to improve separation, such as stepwise synthesis, external force-assisted synthesis, and the surface modification of supports. Huang et al. prepared a dense zeolite membrane by connecting an LTA zeolite membrane with a support through various covalent linkers, such as 1,4-diisocyanate (DIC-4), 3-chlropropylyrimethoxysilane (CPTMS), and 3-amino propyltriethoxysilane (APTES) (Figure 4) [88,89,90]. The covalent linker caused a densification of the LTA membrane and improved the separation efficiency for H_2_/N_2_, H_2_/CO_2_, and H_2_/CH_4_ separations.

Despite all the developments and modifications, zeolite membranes are associated with critical disadvantages, i.e., complex preparation methods and degradation in high-temperature, acidic, or basic conditions. The transformation of zeolite membranes from a lab-scale to an industrial-scale development and utilization presents a huge gap. Their commercial application is still at the developmental level. They have high efficiency and selectivity due to their molecular sieving ability, but the large-scale production of defect-free zeolite membranes is very expensive due to the need for precise control over the crystal growth and the need for a highly stable support. Therefore, maintaining quality and performance at an industrial scale is challenging.

### 2.3. Silica Membranes

Silica membranes were projected as an alternative to metallic membranes to counter their limitations for H_2_ purification. Silica membranes are advantageous in terms of manufacturing costs and chemical and hydrothermal stabilities [91,92,93]. As silica membranes are not composed of any precious metals, they are cost-effective, and the absence of metals makes them immune to hydrogen embrittlement and poison [93]. Silica membranes have a micropore network of ~0.5 nm in diameter, which facilitates the selective permeation of hydrogen-like smaller molecules and omits CO, CO_2_, O_2_, and N_2_, which are of comparatively larger sizes [94,95].

As the silica membrane is composed of a network of micropores, these membranes are not as selective as ceramic membranes, dense metal, and metal alloy membranes. The hydrogen separation is facilitated by a coalition of surface diffusion and molecular sieving. Comparatively smaller H_2_ molecules travel through the network of connected micropores by a site-hopping diffusion mechanism [96,97]. Hence, this is a size-based selective separation facilitated by a pressure difference.

**Figure 5:**
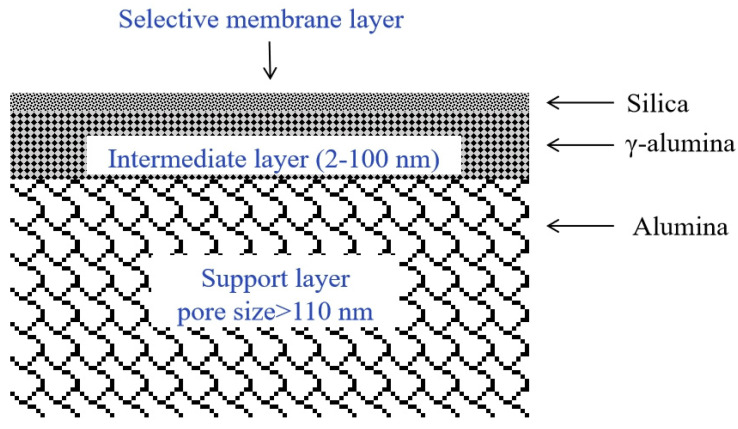
Schematic representation of composite silica membrane.

**Figure 6:**
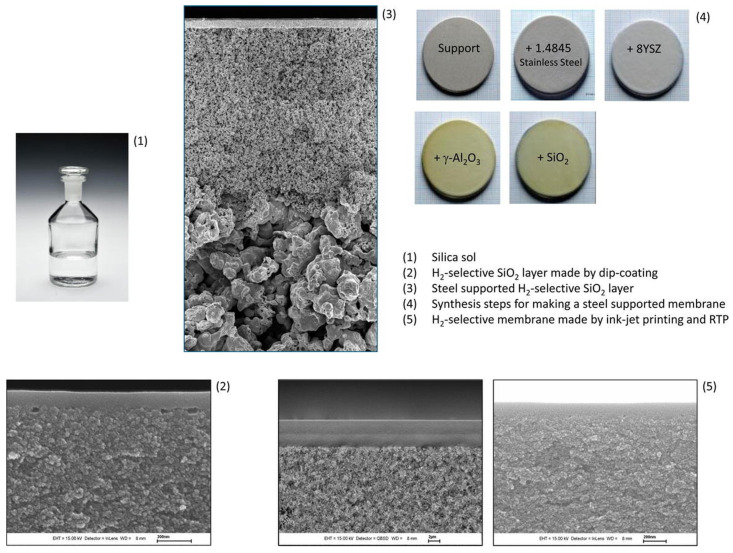
Preparation of stainless-steel-supported silica membranes and their SEM images. Reprinted with permission from ref. [101].

Silica membranes are composed of three layers: the selective membrane layer of silica, the intermediate layer, and the support layer (Figure 5). The selective layer is fabricated as a very thin film of silica on the porous support, while the intermediate layer is prepared from γ-alumina and the support prepared from α-alumina [98]. Alumina supports with a pore size of >110 nm are suitable for industrial utilization due to their high-temperature resistance, strength, and lower cost compared to other supports. An intermediate layer of γ-alumina (2–100 nm) is used between the selective silica layer and the support layer (α-alumina) [93,99,100]. In some reports, stainless-steel metal supports were also utilized (Figure 6) [101]. Metal supports improved the mechanical strength and stability, but due to their fabrication issues, metal supports are not widely utilized.

Silica membranes are generally prepared by the sol–gel method and CVD. In the sol–gel route, the polymeric sol–gel method is most widely employed. Silica polymers are prepared by the hydrolysis and condensation of tetraethyloxosilane (TEOS) (Alkoxysilane). First, hydroxyl groups exchange alkoxide groups (hydrolysis) and by a condensation reaction, silanol groups form siloxane bonds. Furthermore, the silica polymer sol–gel solution is applied onto the mesoporous support by dip coating. The coated support is further dried and calcinated [102,103]. The prepared silica membrane has a top selective layer with pore size of ~0.5–0.8 nm and a thickness of ~50–100 nm [93].

In the chemical vapor deposition method, the pores of the support layer are modified by coating a thin layer through a gaseous reaction at high temperatures. Tetramethoxy silane and tetraethoxy silane, along with nitrogen or argon, are used to deposit a thin layer of silica by CVD [93]. Compared to the sol–gel method, CVD-based silica membranes exhibit high H_2_ permselectivity.

CVD is carried out by two methods, which differ in the mode of contact between support pores and precursors. In the first approach, reactants are introduced from one side of the porous support and are vacuumed from the opposite side. In the other approach, both reactants are counter-contacted by flowing from opposite sides toward each other to create a thin SiO_2_ film [104]. Controlling the pore size and its characterization is difficult due to the sub-nano level at which this process occurs. However, there are several reports on the successful control of the pore size. Otha et al. investigated the effects of the number of phenyl groups on membrane performance. The pore size and gas permeance are enhanced, with an increase in the number of phenyl groups in the reactants, including TMOS (tetramethoxysilane), PTMS (phenyltrimethoxysilane), and DMPS (dimethoxydiphenylsilane) [105]. Silica membranes prepared by the sol–gel method show higher permeability and selectivity, while in the case of membranes prepared by CVD, these exhibit high selectivity but low permeability. The sol–gel method is economical and simple but difficult to reproduce [93]. On the other hand, the CVD technique involves huge capital costs and needs to be conducted under controlled conditions.

Despite all these advantages, hydrothermal instability is the biggest concern. The moist gas feed stream introduced at high temperatures alters the membrane structure, which further reduces the membrane performance and H_2_ flux. This reduction in permeability is due to degradation, also called densification. Densification occurs in four successive steps: (i) water uptake on the pore surface through silanol groups, (ii) the cleavage of siloxane bonds to form additional silanol groups, (iii) the movement of silica oligomers in the pore, and (iv) the condensation of silanol groups to form a dense silica structure. Densification causes the collapse of small pores and the enlargement of large pores, leading to a reduction in permeability and selectivity and the breakdown of the selective silica layer. As steam is a common constituent in H_2_ manufacturing processes, this issue is a setback in the utilization of silica membranes for industrial H_2_ production [106,107,108,109].

**Figure 7:**
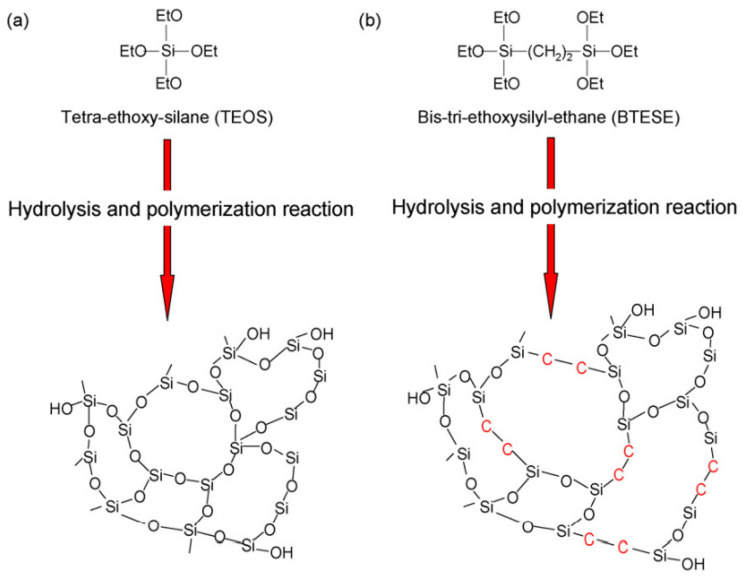
Schematic representation of amorphous silica networks derived by TEOS (**a**) and BTESE (**b**). Reprinted with permission from ref. [110].

Hydrothermal instability can be minimized by enhancing the hydrophobic character of silica membranes. Kanezashi et al. prepared bis(triethoxysilyl) ethane (BTESE)-derived silica membranes, which exhibited improved hydrothermal stability because of the Si–C–C–Si bonds present in the silica network. This improved the hydrophobicity, which further reduced Si-OH formation in hydrothermal conditions. Figure 7 represents the amorphous silica networks derived by TEOS (a) and BTESE (b) [110]. Vos et al. reported on the addition of MTES (methyltriethoxysilane) to a TEOS (tetraethylorthosilicate) solution. The introduction of the -CH_3_ group significantly improved the membrane hydrophobicity by a factor of 10 [111]. The addition of various transition metals, such as ZrO_2_, NiO, Co_3_O_4_, Al_2_O_3_, Nb_5_O_2_, Y_2_O_3_, and TiO_2_, as dopants significantly improves hydrophobicity, selectivity, and the H_2_ flux [47,112,113]. Therefore, composite silica membranes with hybrid membrane materials are more promising in terms of permeability, selectivity, and mechanical strength.

Silica membranes are inexpensive, but their scalability and commercialization are limited by the complexity in ensuring a uniform pore size and cracking during operation. For their advancement, composite membranes are prepared with metals and silica to improve strength and reduce fragility. As they have moderate H_2_ selectivity and low costs, continued research and development may lead to their commercialization in the future.

### 2.4. Carbon Molecular Sieve Membranes

Carbon membranes are prepared by pyrolysis or the carbonization of various precursor materials, mostly organic polymers, which, upon pyrolysis, are converted to amorphous carbon [114]. The structural properties (porosity and pore dimension) of amorphous carbon can be adjusted by varying the process parameters. The high chemical and thermal resistance, tunable pore-size distribution, and high selectivity of CMSMs make them significant in gas separation [115]. The H_2_ selectivity of CMSMs is comparatively higher than zeolite membranes; however, it is much lower than dense membranes [47]. Rao and Sircar analyzed a nonporous selective surface flow (SSF) membrane prepared by the carbonization of PVC. These authors found that it has a comparatively bigger pore size (0.5–0.7 nm) than that of CMSMs [116].

Depending on their application, CMSMs can be prepared in both supported and unsupported forms [114]. Both are associated with the problem of brittleness. To counter this issue, multiple cycles of polymer coating and carbonization can be performed to obtain defect-free membranes [117]. These membranes can have different configurations. Supported carbon membranes have a flat and tube form, while unsupported ones have a flat-sheet, capillary, or hollow-fiber configuration [118]. Supported membranes are mostly preferred due to their comparatively better mechanical strength over unsupported membranes. In membrane preparation, for supported membranes, there are several options for coating the support with polymeric films, for instance, spin coating, ultrasonic deposition, vapor deposition, spray coating, and dip coating [119].

The development of a carbon-based membrane consists of several successive steps of fabrication and modification: (i) precursor material selection, (ii) the preparation of precursor material, (iii) pretreatment, (iv) pyrolysis, (v) post-treatment, and (vi) module fabrication [120]. Among these, material selection and pyrolysis are the most critical steps, as they determine the pore dimensions and performance.

Precursor selection: Various carbon materials, such as, coal, plants, pitch, resins, graphite, and numerous synthetic polymers, have been pyrolyzed to prepare carbon membranes [120]. Generally, preference is given to polymeric precursors because of their thermosetting properties, high yield of fixed carbon, good processability, and homogeneous texture [121]. Polyimides, poly(furfuryl alcohol) (PFA), polyetherimides (PEIs), polyacrylonitrile (PAN), polyvinylidenechloride-acrylate terpolymer (PVDC-AC), and phenolic resins are the most widely used precursors [122,123,124]. The polymer solution is prepared in the appropriate solvent, and the membrane is further prepared by spin coating, dip coating, or film casting. The selected polymer should be such that it does not soften or melt under pyrolysis and is not prone to the formation of pinholes and cracks in the carbon structure. Additionally, when selecting the support, some important criteria, such as compatibility, heat transfer ability, durability, chemical reactivity, and the cost of the support, must be considered [125].

Pretreatment: Physical and chemical pretreatments are conducted prior to pyrolysis. Chemical pretreatments are applied to modify and tailor the essential characteristics. Oxidation pretreatments are the most widely used technique capable of stabilizing precursor polymeric materials, thereby protecting the membrane from melting and forbidding the excessive emission of volatile gases during the pyrolysis process. Physical treatment involves the stretching of the membrane prior to pyrolysis [118,126].

Pyrolysis is the most important step in membrane development, involving the heating of precursor material under controlled conditions with a specific protocol to obtain an amorphous microporous carbon membrane with the desired configuration. The pore size, structure, interconnectivity, and membrane performance are directly related to the precursor material and pyrolysis-process parameters [118,127].

Post-treatment is applied for the fine-tuning of the pore size, shape, and distribution to achieve the desired separation. Several post-treatment methods have been reported for the improvement of carbon membranes, including post-pyrolysis, post-oxidation, coating, and chemical vapor deposition (CVD) [118,126].

The *module design* depends on various factors, such as the cost of the membrane, industrial requirements, and separation efficiency. The hollow-fiber configuration is most preferred due to its high permeability and selectivity. Lagorsse et al. analyzed hollow fiber and flat-sheet carbon molecular sieve membranes in the honeycomb confirmation [128,129]. In the end, the cost and applicational requirements of the membrane module govern the final design.

Challenges with CMSMs: The lack of reproducibility, brittleness, and high manufacturing cost are the major issues associated with CMSMs. They are three times costlier than polymer membranes [130]. Furthermore, the presence of oxygen and humidity hinders membrane performance. In the presence of air, O_2_ binds with the active sites of CMSM and creates functional groups with the surface oxygen, which decreases porosity. During the interaction with moisture later on, the water is adsorbed on hydrophilic sites. After the initial adsorption, more and more water molecules are attached via hydrogen bonds. The formed water cluster blocks the CMSM membrane [131].

**Figure 8:**
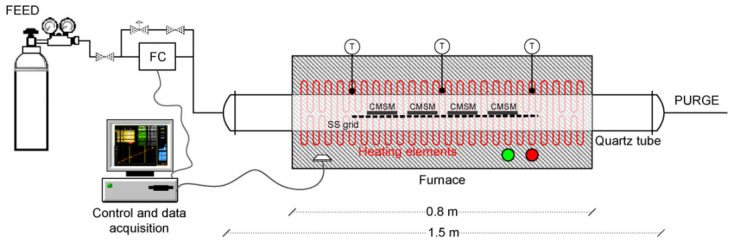
Schematic representation of the pyrolysis setup. Reprinted with permission from ref. [132].

To overcome the issues associated with humidity, Campo et al. developed membranes from cellophane paper. Three different membranes were prepared by varying the soaking times, abbreviated as Celo550-ST60, Celo550-ST240, and Celo550-ST480. Figure 8 represents the pyrolysis setup utilized to prepare a series of membranes. The membranes showed no aging effects in the presence of humidity and O_2_. The cellophane paper-based CMSMs exhibited permselectivity beyond the Robeson upper bound [132].

Composite CMSMs have been prepared to eliminate the shortcomings associated with CMSMs, such as their lack of reproducibility, brittleness, and low mechanical strength, and to enhance H_2_ selectivity and permeability [119]. Mixed-matrix membranes with inorganic particles (zeolites, metals, carbons, and silica) are pyrolyzed to obtain composite CMSMs. Sazali et al. discussed various MMMs with improved H_2_ permselectivity [133]. Carbon nanotubes (CNTs) are also promising materials for H_2_ purification. The smooth walls of CNTs facilitate high permeability. Ge et al. developed a vertically aligned CNT-supported zeolite membrane. The zeolite imidazole framework (ZIF) was grown on the VACNT membrane by secondary seeded growth. The ZIF layer acted as a selective layer and exhibited higher H_2_ selectivity [134].

Carbon molecular sieve membranes (CMSMs) are commercially viable due to their high selectivity, excellent thermal stability, and tunable pore sizes, making them well-suited for hydrogen separation. However, their scalability is limited by the need for a precise process control during fabrication to ensure the formation of appropriate pore sizes. The fabrication process, which typically involves carbonization or pyrolysis, requires the careful control of parameters such as the temperature and atmosphere.

To overcome these scalability challenges, the selection of appropriate precursors and the optimization of the pyrolysis process are critical. By choosing suitable polymer precursors and by fine-tuning the carbonization conditions, it is possible to produce CMSMs with the desired pore-size distribution and with enhanced mechanical properties. This can significantly improve the scalability of CMSMs while maintaining their high performance for hydrogen separation.

## 3. Polymeric Membranes for H_2_ Separation

Polymeric membranes have a broad spectrum of applications in the field of gas separation, such as flue gas separation, biogas separation, oxygen purification, hydrogen purification, etc. Polymeric membrane technology features several benefits over conventional membranes in terms of a lower energy consumption, low cost, ease of handling, ease of scale-up, low environmental impact, and ease of combination with other technologies for improved separation. Polymeric membranes are mainly categorized as (i) glassy polymers and (ii) rubbery polymers. The most widely studied polymers are as follows: with respect to glassy polymers, these include cellulose acetate (CA), polysulfone (PSF), polyimides (PIs), polycarbonates (PCs), poly(phenylene oxide), and polyperfluorodioxoles, and with respect to rubbery polymers, these include poly(dimethylsiloxane) (PDMS) and ethylene oxide/propylene oxide–amide copolymers [135]. The comparatively higher free volumes in glassy-polymer membranes favor the transportation of gases through their voids [136]. The ideal candidate for polymeric membranes should have high chemical stability, mechanical strength, and thermal stability, along with high permselectivity. The limiting parameter for most polymeric membranes is the trade-off between permeability and selectivity, which is described as Robeson’s upper bound. Thus, a membrane with high permeability shows low selectivity and a high-selectivity membrane exhibits low permeability [46]. Based on the porosity or free volume, membranes can be further distinguished as dense nonporous and porous membranes. Porous membranes have a greater free volume due to their randomly distributed pore network. Here, the separation occurs through a sieving mechanism based on the size of pores and molecules. A nonporous membrane is a dense polymeric film which allows for gas permeation to occur via adsorption and diffusion. This diffusion is facilitated by driving forces such as the electrical potential gradient, concentration difference, or pressure difference. The gas transport through the membrane depends on its solubility and diffusivity through the membrane material [137]. The gas transport through the membrane follows various mechanisms, as described below.

**Figure 9:**
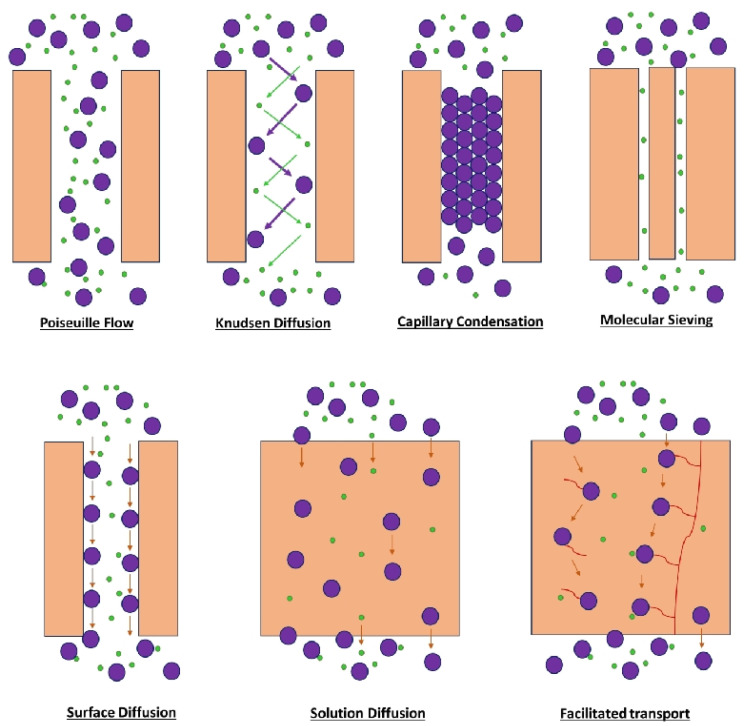
Schematic representation of various gas transport mechanisms.

Figure 9 shows the various gas transport mechanisms through the membrane, e.g., the Poiseuille flow, Knudsen diffusion, capillary condensation, molecular sieving, surface diffusion, and facilitated transport. In the Poiseuille flow mechanism, gas separation occurs depending on their differential flow rates through the membrane. The flow of gases is governed by the molecular size of the gases, pore radius, pore length, pressure difference, and the viscosity and diffusivity of the gas material [138]. Knudsen diffusion takes place when the membrane pore size is smaller compared to the mean free path. Hence, the gas molecules repeatedly collide with the pore wall and pass the membrane. This involves greater molecule–wall collisions than intermolecular collisions. The gases are separated based on their molecular velocity difference. Molecules with a low molecular weight (H_2_) travel faster than heavier gases such as CH_4_ and CO_2_. For Knudsen diffusion, the membrane should be finely porous with a controlled pore size [139].

The capillary condensation mechanism takes place when one of the gas components from the mixture is condensable. At a lower pressure, the gas condenses into the small pores of the membrane. The gas separation occurs based on the difference in condensation properties. The condensed gas occupies the pores, particularly mesopores and small macropores, at a lower pressure than the bulk pressure. Because of the condensation menisci formed at both ends of the pore, transport occurs via a hydrodynamic flow driven by a capillary-pressure difference between the ends. The condensable gas will form a liquid layer, which restricts the flow of non-condensable gases. Gases with an equal molecular size but different condensation properties can be easily distinguished. Hence, capillary condensation provides very high selectivity compared to other mechanisms. As it operates at a lower pressure, it consumes less energy. Here, by varying the polymer membrane material and adjusting the pore structure, tailor-made separation properties can be achieved for particular gases [140].

Molecular sieving occurs if the membrane pore dimension is identical to the required gas component. Smaller-sized gas components can pass through the pore channels, while larger gas molecules will be rejected. Molecular sieving works on the principle of size exclusion. The size or dimensions of the gas molecules can be defined by their kinetic diameter. Thus, the kinetic diameter will be a decisive parameter for separation across a membrane [139]. When a gas molecule is adsorbed on the porous wall of the membrane and migrates along the surface from one end to another, this process is called surface diffusion. For surface diffusion, one of the gas components needs to be preferentially adsorbed onto the membrane surface. The amount of the gas transported through surface diffusion largely depends on the adsorption and movement of the selective component. The driving force may be a difference in the temperature, pressure, or concentration. The migration over the surface can be influenced by the physical and chemical properties of the membrane surface and the interaction between the gas–membrane surface. A high surface area and a high concentration of the selective component make this diffusion dominant over other transport mechanisms [45].

The solution diffusion mechanism is driven by the solubility and diffusion of individual gas components in the polymer matrix [141]. The facilitated transport mechanism occurs through a chemical interaction between the selective component of the membrane and the gas of interest. Here, the selective component of the membrane works as a carrier for the selective gas, transporting the gas of interest through the membrane and inhibiting the travel of other components from the mixture [142].

**Figure 10:**
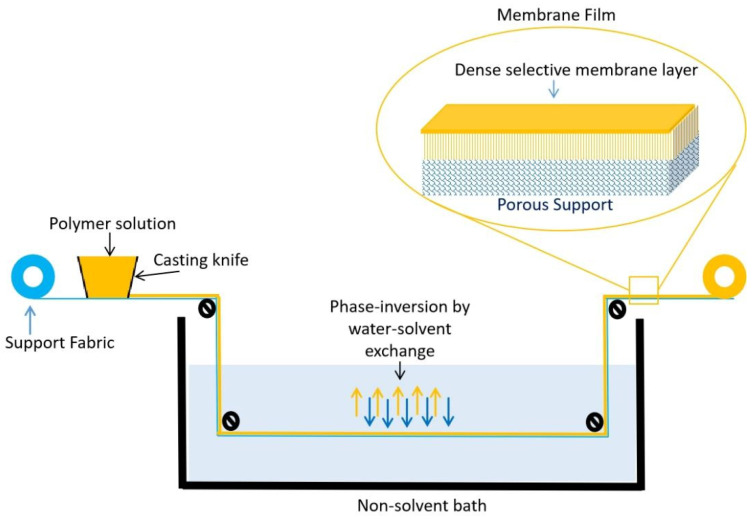
Representation for membrane preparation by the non-solvent-induced phase-inversion process.

**Figure 11:**
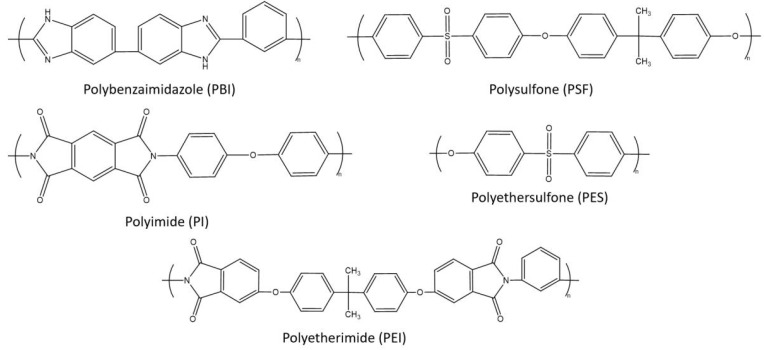
Structures of various promising polymers used for the fabrication of polymeric membranes.

Organic polymers are the primary materials for the preparation of gas-separation membranes due to their high selectivity and ease of processing. Polymeric membranes are generally prepared by the phase inversion process (Figure 10). This process facilitates the fabrication of a large-scale membrane. The prepared membrane has an asymmetric structure, with a thin selective-membrane layer on top of a porous structure [143]. The characteristics of the selective layer and beneath the porous structure depend on the type of polymer used, the solution concentration, the used non-solvent, the temperature, the humidity, the rate of phase inversion, etc. Polymeric membranes are fabricated in various configurations, such as flat sheets, hollow fibers, disk shapes, etc., and their modules are prepared in the form of hollow-fiber tubular modules or spiral-wound membranes [143]. Gas separation occurs through the solution diffusion mechanism. Depending on the affinity toward the polymer material, the membrane can be either a hydrogen-selective or a hydrogen-rejective membrane. These terms will be elaborated on later on in this review. Over time, various polymers, such as polybenzimidazole (PBI), polyimide (PI), polysulfone (PSF), polyetherimide (PEI), and polyethersulfone (PES), were studied for hydrogen purification (Figure 11). Among these, PI, PBI, and their derivatives (Kapton, LARC-TPI, IP-2080, and Matrimid) are the most widely studied due to their high selectivity and thermal stability [45]. However, very few polymers are currently being utilized for industrial operations. Table 1 provides a list of the polymers that are industrially important for gas-separation applications [144,145,146].

The membranes prepared from traditional polymers still have very poor performance in terms of permeability and selectivity. Hence, various strategies have been implemented to improve the performance of polymeric membranes, such as modifications in polymer chain packing, the development of new polymers such as PIMs (Polymers of Intrinsic Microporosity), chemical cross-linking, and polymer blending to achieve benefits from both polymers and design application-suitable membranes. A tremendous amount of research has been conducted on the development and improvement of polymeric membranes. Polymeric membranes are also used as protective and selective layer in hydrogen sensors. For the implementation of the hydrogen economy, hydrogen sensors are also critically important to ensure workplace safety during H_2_ production, purification, storage, and application. However, the H_2_ sensors are vulnerable to deactivation of active sites by poisonous gases, affecting their accuracy, effectiveness and stability. To overcome the limitations, PMMA-based membranes have been fabricated on H_2_ sensors to eliminate poisonous gases [147,148]. The membrane layer provides long-term stability. In the following section, various polymeric membranes from the literature are sorted and presented as H_2_/CO_2_-, H_2_/CH_4_-, and H_2_/N_2_-selective polymeric membranes.

### 3.1. H_2_/CO_2_-Selective Membrane

**Figure 12:**
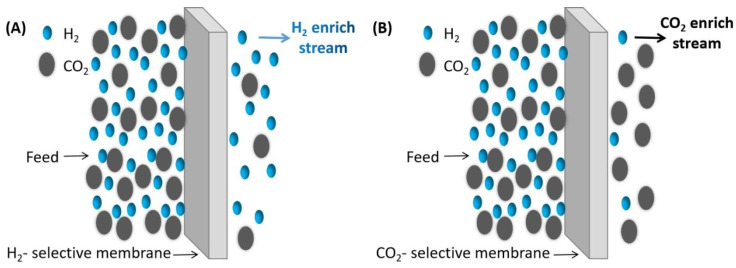
Schematic representation of (**A**) H_2_-selective polymer membranes and (**B**) CO_2_-selective polymer membranes.

H_2_/CO_2_ separation is a part of various industrial processes, where H_2_ is selectively isolated from a gas mixture having H_2_ and CO_2_ as the major components. Two different kinds of membranes might be used, either H_2_-selective membranes or CO_2_-selective membranes. H_2_-selective ones will produce high-pressure CO_2_ and low-pressure H_2_. Here, the retaining gases contain CO_2_ and other contaminants (CH_4_ and CO). Conversely, CO_2_-selective membranes produce high-pressure H_2_ and low-pressure CO_2_ (Figure 12). The majority of reported H_2_/CO_2_ separation membranes are H_2_-selective membranes developed for the enrichment of hydrogen.

There are several reports on polymeric membranes for H_2_/CO_2_ separation. Orme et al. examined various polymers for H_2_/CO_2_ separation. They sought to identify the polymeric membrane which selectively favors hydrogen permeability over CO_2_ and chlorinated organics. Various polymers, such as PSF, PS, PVDF, PDMS, and PMMA, displayed good results at 30 °C. In this study, the prepared membranes were analyzed by individual- and mixed-gas permeability tests. Polystyrene exhibited superior results with high permselectivity [146]. Cong et al. used melamine and pyromellitic dianhydride monomers to construct a network of polyimides through interfacial polymerization. A membrane was employed for the separation of hot H_2_/CO_2_. Upon treatment with steam and H_2_S, the membrane exhibited a H_2_ permeability of 42.3 GPU and a H_2_/CO_2_ selectivity of 18.7 at 623 K and 1 bar [149].

**Figure 13:**
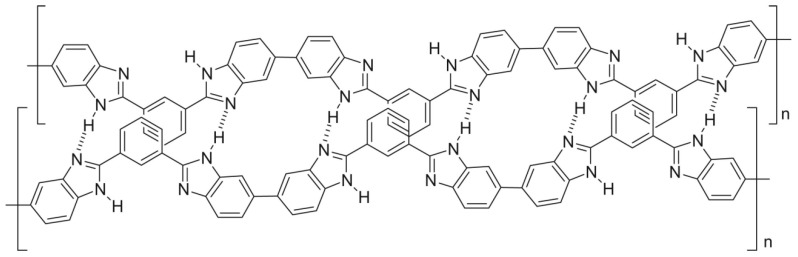
Representation of PBI structural packing: π–π stacking and hydrogen bonding. Reprinted with permission from ref. [152].

Matrimid^®^, a polyimide, has high thermal and chemical stability and high H_2_ selectivity and permeability, making it a key material for the development of hydrogen purification membranes [150]. Favvas et al. prepared a carbon membrane from a Matrimid^®^ 5218 polyimide hollow-fiber precursor. The prepared membrane had a higher pore volume, higher microporosity, and a high absorption area. However, after the pyrolysis process, the membrane retained its asymmetric structure, and the resulting carbon membrane showed a very good H_2_ permeance of 20 to 52 GPU with a H_2_/CO_2_ selectivity of 37.8 [151]. H_2_/CO_2_ selectivity of polymers can be effectively increased by decreasing the free volume and increasing the molecular-sieving capabilities. Various strategies have been investigated to reduce the free volume, either by polymer blending or cross-linking. Polybenzimidazole (PBI) and its derivatives, with a high chain-packing density, are promising candidates for H_2_/CO_2_ separation. Their effective chain packing and low free volume are due to π–π stacking and hydrogen bonding (Figure 13) [152].

**Figure 14:**
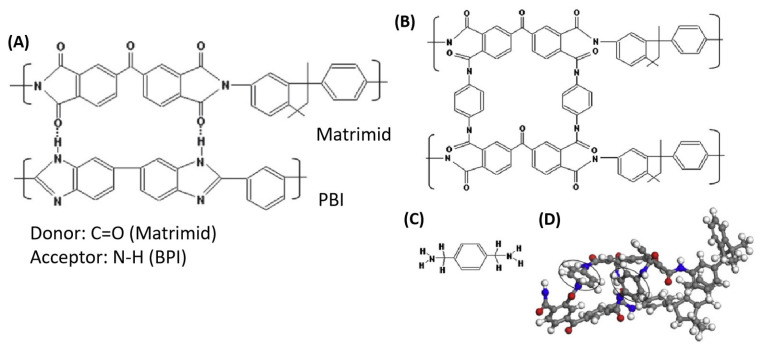
(**A**) Schematic representation of the chemical structure of polymers and hydrogen-bonding interactions between the functional groups of Matrimid and PBI. (**B**) Proposed mechanism for the chemical cross-linking modification of the Matrimid component of the blend using p-xylene diamine. (**C**) Chemical structure of p-xylene diamine and (**D**) possible chain morphology and configuration of p-xylene diamine cross-linked with Matrimid (cross-linking agents are specified by ovals). Reprinted with permission from ref. [153].

Hosseini et al. prepared blend membranes from Matrimid^®^ and PBI. This blend demonstrated higher miscibility due to the strong hydrogen-bond interactions between the functionalities of the blending component, PBI, and Matrimid^®^, which improved the chain-packing density and hindered the polymer chain mobility, resulting in an overall improvement in the gas-separation performance. The Matrimid^®^/PBI (25/75%) blended membrane cross-linked with p-xylene diamine showed a H_2_/CO_2_ selectivity of 26. The same blended membrane showed an excellent H_2_/N_2_ separation of 271 (Figure 14) [153].

**Figure 15:**
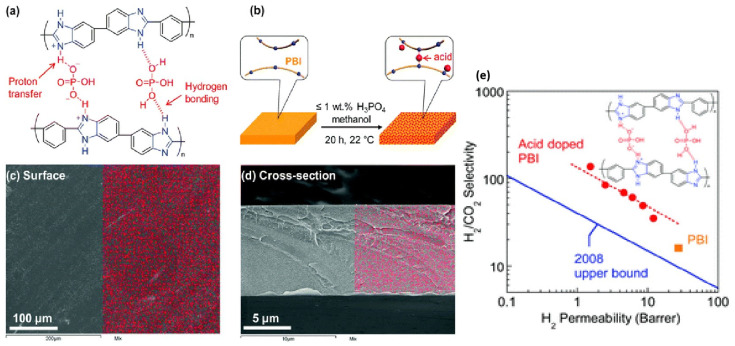
Schematic illustration of (**a**) proton transfer mechanism and hydrogen bonding in the PBI–H_3_PO_4_ complex and (**b**) the preparation of H_3_PO_4_-doped PBI films with PBI backbones cross-linked by acids. SEM images with an overlaid SEM/EDS mapping of phosphorus on the (**c**) surface and (**d**) cross-section of a PBI–(H_3_PO_4_) 1.0 film. The red dots display the distribution of phosphorus in the polymer, (**e**) the comparative permselectivity of the acid-doped PBI. Reprinted with permission from ref. [154].

Zhu et al. used a PBI membrane doped with polyprotic acids to enhance the H_2_/CO_2_ selectivity. PBI was doped with polyprotic acids such as H_3_PO_4_ and H_2_SO_4_, which cross-linked the PBI polymer chains and decreased the free volume. The resulting membrane showed excellent stability up to 200 °C and a H_2_/CO_2_ selectivity of 140, even at 150 °C, which is superior to the known polymer materials. The size-sieving capability and H_2_/CO_2_ separation capabilities of PBI improved by modifying the chain packing through doping with polyprotic acids followed by cross-linking (Figure 15) [154].

### 3.2. H_2_/CH_4_-Selective Membrane

Hydrogen separation from methane is critical in various industrial processes, such as hydrogen purification for applications in fuel-cell, biogas, and natural gas upgrading. Advancements in membrane materials and technology have led to the development of several polymeric membranes providing promising results in H_2_/CH_4_-selective separation.

**Figure 16:**
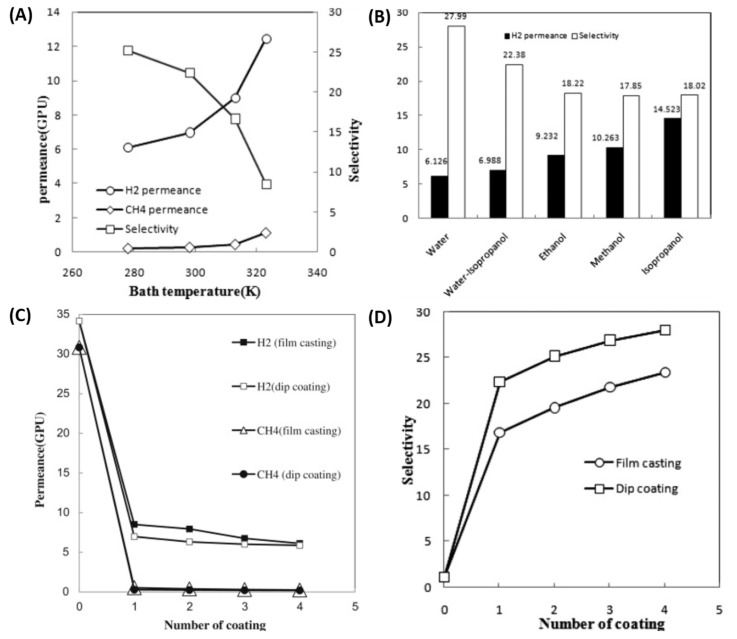
(**A**) Effects of the coagulation bath temperature on real selectivity and gas permeance. (**B**) Effects of different non-solvents on selectivity and H_2_ permeance. (**C**) Effects of sequential coating on permeance. (**D**) Effects of sequential coating on the selectivity of hydrogen/methane binary mixture with a 50–50% concentration at 1 bar and 25 °C for film casting and dip coating. Reprinted with permission from ref. [155].

**Figure 17:**
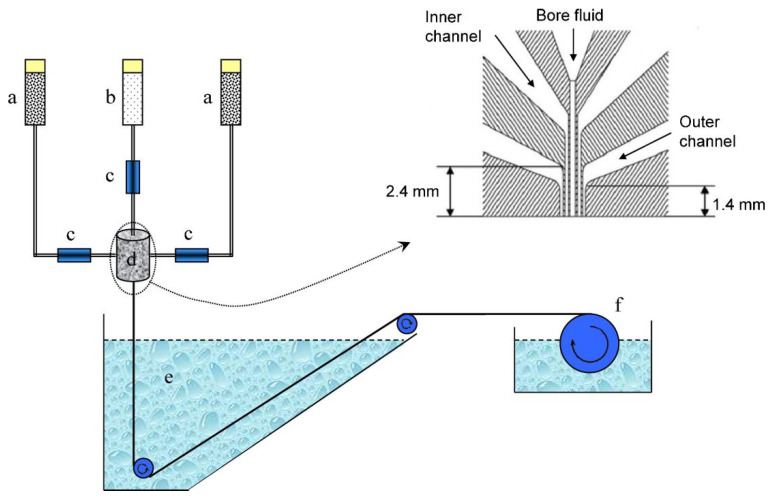
Schematics of dual-layered hollow-fiber spinning setup and triple-orifice spinneret: (a) dope fluid tank and pump; (b) bore fluid tank and pump; (c) filter; (d) spinneret; (e) coagulation bath; and (f) take-up drum. Reprinted with permission from ref. [157].

Shamshabadi et al. prepared a H_2_/CH_4_-selective PDMS/PEI composite membrane by coating a PDMS layer on the asymmetric PEI support. PDMS coating was performed by film casting and dip coating. The effects of the non-solvent bath temperature on the membrane structure were also investigated. This study revealed that by increasing the temperature, the permeability increases but the selectivity decreases, while increasing the pressure improves selectivity and reduces permeability. Lowering the coagulation bath temperature makes the membrane denser and increases selectivity. The effects of non-solvents on the membrane morphology are explained by the precipitation time and solubility parameters. Water results in a denser membrane due to the shorter precipitation time, while isopropanol forms a sponge-like structure. Dip-coated membranes have higher selectivity than film-cast ones due to their better coating coverage and the PDMS penetration into pores. Sequential coatings increase selectivity by sealing uncovered pores and defects. Sequential coating with different concentrations increases selectivity from 22 to over 70 (Figure 16) [155]. Kargari et al. also investigated PDMS/PEI composite membranes for H_2_/CH_4_ separation. The PDMS coating conditions were optimized by applying three different coating methods: film casting, pouring the solution at a 45° angle, and dip coating. The effects of the solution concentration, curing temperature, and successive coating on the permeability and selectivity were studied. The results show that PDMS concentration of 15 wt.% is sufficient, while the coating and curing temperature do not have much of an effect on H_2_ selectivity and permeability. Successive dip coating exhibited promising results due to the penetration of the PDMS coating solution into the pores of the membrane surface. Successive dip coating improved the H_2_/CH_4_ selectivity from 25 to 96 (for the pure gas) and from 22 to 70 (for the binary mixed gas) at a 1-bar feed pressure and 25 °C [156]. Hosseini et al. prepared a dual-layer hollow fiber from a PBI–Matrimid (1:1) interpenetrating network polymer blend. The membrane was coated with 2% Sylgard-184 (silicon rubber) to cover the defects. Figure 17 shows a schematic representation of the fabrication of dual-layered hollow-fiber membranes through a dry-jet wet spinning process. Further chemical modifications were performed with 10% of p-xylylenediamine in methanol. The resulting membranes were delamination-free at the interface and had favorable morphological and microstructural properties, especially at the outer functional layer. An analysis of the air–gap distance and elongation showed that both increase membrane permeability. The results show that spinning at a 3 cm air–gap distance can produce membranes with a H_2_/CH_4_ selectivity of 89.20 and a H_2_/CO_2_ selectivity as high as 11.11. The prepared membrane also showed very high resistance values against CO_2_-induced plasticization [157].

Shishatskiy et al. developed asymmetric polyimide membranes for hydrogen separation. They studied the effects of variations in casting solutions and membrane preparation methods for a pilot-scale membrane fabrication. The findings indicate that H_2_/CH_4_ selectivity is roughly 100, which is close to the selectivity of dense the Matrimid 5218 membrane. The non-woven fabric used in the fabrication provided exceptional mechanical strength to the membrane. Additionally, the membrane exhibited high thermal stability up to 200 °C. Furthermore, PDMS surface coating was once again proven to be an effective way to treat surface defects of the selective layer [158]. Some key polymers, such as polyimide and its derivatives, with their H_2_ permeability and H_2_/CH_4_ selectivity are presented in Table 2. A corresponding table with additional information for the presented membranes (Table 2) is provided in the supporting information as Table S2.

### 3.3. H_2_/N_2_-Selective Membrane

H_2_/N_2_ separation is vital in various processes, such as fuel-cell applications, refinery operations, and ammonia manufacturing. These membranes are engineered by considering the physiochemical characteristics of H_2_ and N_2_ to enrich high-purity H_2_.

Rezac et al. blended commercial polyetherimide (PEI) Ultem^TM^ with an acetylene-terminated (ATM) monomer to investigate the resulting thermal and transport properties. The blending of ATM decreased the glass transition temperature (Tg) and gas transport. Furthermore, PEI was blended with un-crosslinked and crosslinked monomers. The results show that the crosslinked additives increased the thermal and chemical stability along with the gas permeability and selectivity of the PEI compared to the un-crosslinked blend. The improved chemical stability of membranes makes them suitable for the isolation of hydrogen from hydrocarbons in harsh chemical environments. By tuning the degree of ATM curing, the chemical resistance of the membrane can be adjusted between a fully crosslinked blend and pure PEI. Among various compositions, a 91% PEI/9% ATM blend cured at 230 °C showed superior H_2_/N_2_ selectivity [164].

**Figure 18:**
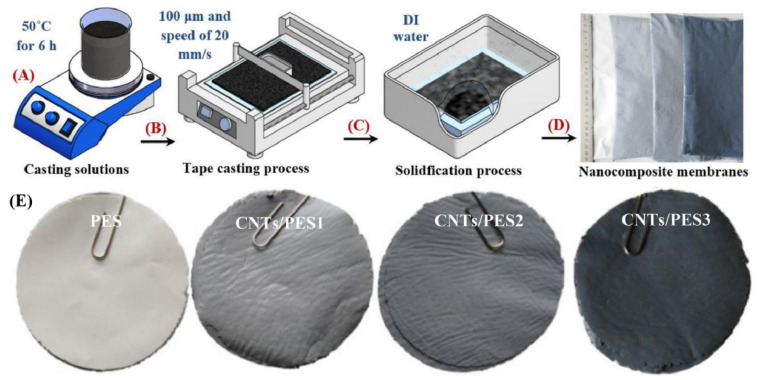
(**A**–**D**) Representation of various steps in the fabrication of CNT/PES membranes. (**E**) Digital images of prepared PES membranes and CNT/PES membranes. Reprinted with permission from ref. [166].

**Figure 19:**
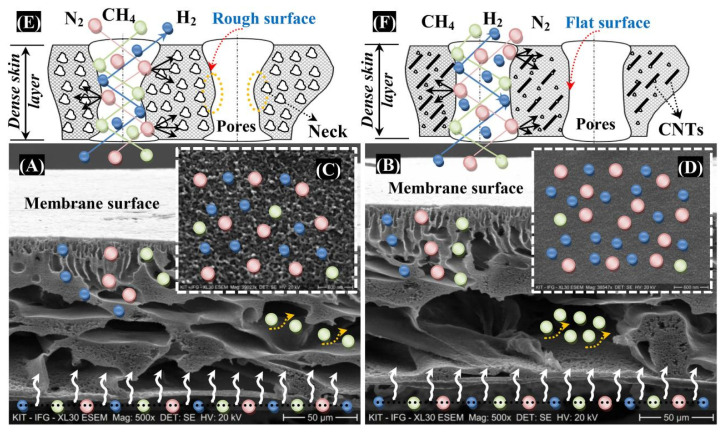
SEM images of (**A**,**C**) PES membrane and (**B**,**D**) CNTs/PES. (**E**,**F**) Schematic representation of pore structure and gas transport mechanism through the PES pore walls and CNT/PES flat-smooth walls. Reprinted with permission from ref. [166].

**Figure 20:**
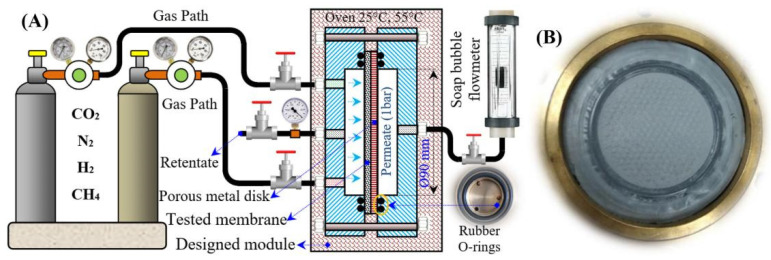
(**A**) Schematic representation of the experimental setup utilized to measure the permeability of membrane gases. (**B**) Digital image of CNT/PES membranes after a gas separation experiment. Reprinted with permission from ref. [166].

Bernardo et al. studied the effects of a post-spinning solvent exchange on the performance of polyimide hollow-fiber membranes. They prepared the membranes with a conventional and triple-orifice spinneret, followed by solvent exchange with various alcohols (MeOH, EtOH, isopropanol, *tert*-butanol, and 1-butanol) and then alcohols/n-hexane. The alcohol/n-hexane mixture caused damage to the selective layer, which subsequently increased the permeability and reduced the selectivity. The n-hexane caused polymer swelling and destruction of the membrane structure. Conversely, tert-butanol improved the permeability without compromising selectivity in the hollow fibers spun by the triple-orifice spinneret. The prepared hollow fiber showed a H_2_ permeance of 40 with a H_2_/N_2_ selectivity of 75.5 at a 25 °C temperature and a 1-bar pressure [165]. Yousef et al. prepared ultra-permeable (CNTs/PES) nanocomposite membranes by blending a very minute concentration of carbon nanotubes (CNTs) (0.01–0.03 wt.%) into polysulfone (PES), denoted as CNTs/PES1, CNTs/PES2, and CNTs/PES3. These membranes were prepared by film casting on a glass plate using an automatic film applicator, followed by phase-inversion in deionized water. Figure 18 represents the fabrication of the membranes and digital images of the prepared membranes. The membranes consisted of a thin dense layer with fine pores and porous layers with high porosity. A chemical analysis revealed the uniform incorporation of CNTs, decreased the crystallinity of the PES, and increased the free space for gas transfer. The membrane porosity increased from (PES) 81.7% to (CNTs/PES3) 88.4%, and the pore size reduced from (PES) 84 nm to (CNTs/PES3) 50 nm. Figure 19 shows the cross-section and surface SEM images of the PES and CNT/PES1 membranes. Both membranes have a dense selective layer and a porous layer. The increased porosity, reduced pore size, and smooth pore walls of CNTs/PES1 improve the permeability. Figure 19E,F shows the comparatively smooth and flat pore walls of CNTs/PES1 compared to the PES membrane, which further facilitated the easy and rapid gas transport. The gas transport occurred through the Knudsen diffusion mechanism. The CNT/PES membranes demonstrated the excellent CH_4_/N_2_ and H_2_/N_2_ selectivity of 1.62 and 3.95 compared to the 0.33 and 0.76 of the PES membranes. Figure 20 represents the experimental setup utilized to measure gas permeability through the prepared CNT/PES membranes. The apparatus is capable of operating under varied gas flow rates and temperatures [166]. These developments in H_2_/CO_2_-, H_2_/CH_4_-, and H_2_/N_2_-selective separation membranes will improve industrial operations and encourage the implementation of a hydrogen economy.

### 3.4. Limitations of Polymeric Membranes

Currently, polymeric membranes are widely used for gas separation due to their thin film processability, mechanical strength, good separation efficiency, low cost, and low environmental impact. However, as mentioned above, polymeric membranes are limited by the trade-off between permeability and selectivity. Swelling, plasticization, thermal stability, and aging are the major challenges associated with polymer membrane materials. Various glassy polymers, like polypropylene oxide (PPO), polysulfone (PSF), and polyimide (PI), as well as polymers that have a high void volume, for example, Hyflon^®^, Teflon^®^ AF, and PIMs, are more susceptible to physical aging. However, various techniques have been employed to minimize aging in polymers, such as membrane post-modifications, polymer backbone modifications, and blending with nonporous materials [49,167]. On the other hand, silica, zeolite, ceramic, carbon, and MOF-based inorganic membranes have higher chemical and thermal stability. These inorganic fillers have well-defined pore structures, which can be further tuned based on the separation requirements. They can easily separate gases based on the molecular size. However, they also suffer from various limitations, such as a lack of reproducibility, high cost, brittleness, and complexity in synthesis and fabrication, which restricts the utilization of inorganic membranes in large-scale industrial processes [168].

Polymeric membranes offer moderate hydrogen selectivity but stand out for their ease of fabrication through methods like phase inversion and solution casting. Their low production costs and scalability make them highly attractive for commercial applications. To counter the challenges associated with inorganic membranes and polymeric membranes, mixed-matrix membranes were proposed as a potential alternative. The cooperative effect of polymer processability and the separation ability of inorganic additives significantly improve the properties and performance of membranes.

## 4. Mixed-Matrix Membranes (MMMs)

**Figure 21:**
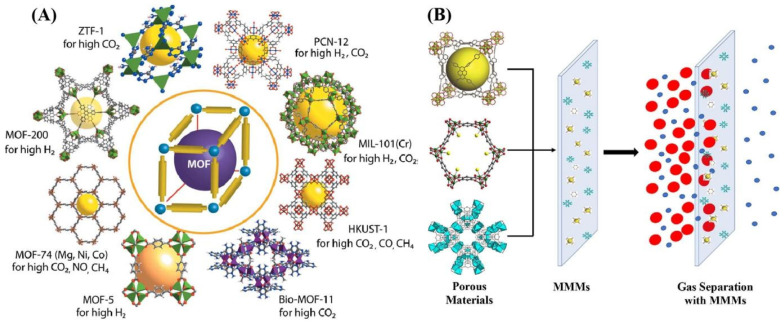
(**A**) Three-dimensional structure of various MOF materials. Reprinted with permission from ref. [179]. (**B**) Schematic representation of porous inorganic filler-based MMMs for gas separation. Reprinted with permission from ref. [168].

MMMs are composed of a continuous polymer matrix with well-dispersed inorganic fillers in the polymer matrix. The presence of an inorganic filler features high selectivity, while the polymer phase enhances the processability, flexibility, and mechanical strength of prepared MMMs [169]. These membranes are capable of resolving the challenges associated with both polymeric and inorganic membranes. These hybrid membranes exhibit high selectivity and permeability, along with a greater mechanical, chemical, and thermal stability and processability [168]. A variety of inorganic fillers, such as MOFs [170,171], COFs [172,173], zeolites [174,175], graphene [176], silica [177], and ZIFs (zeolitic imidazolate frameworks) [178] have been employed to prepare MMMs for H_2_ purification. Figure 21A details the 3D structure of various MOF materials used for gas separation along with porous inorganic filler-based MMMs (Figure 21B) [168,179]. The inorganic filler should have high dispersibility in the matrix, high compatibility with the polymer phase, and exceptional stability under different working conditions [49].

**Figure 22:**
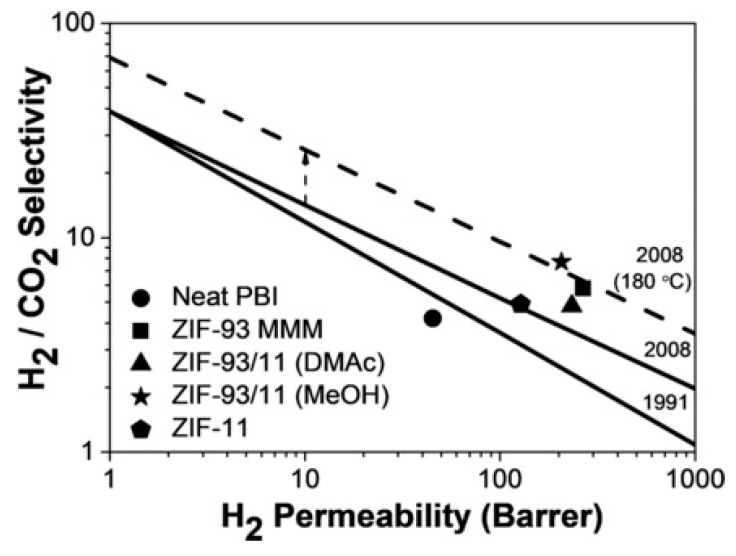
Gas-separation performance of bare PBI membranes and 20 wt.%-loaded MMMs containing ZIF-93, ZIF-11, and the ZIF-93/11 hybrid materials, which were synthesized in DMAc and MeOH. The continuous lines correspond to the original Robeson upper bounds of 1991 and 2008, and the dashed line corresponds to the upper bound calculated for 180 °C. Reprinted with permission from ref. [180].

Sánchez-Laínez et al. developed a nano-sized hybrid ZIF by the post-synthetic modification of ZIF-93 in a benzimidazole (bIm) solution. The addition of bIm enhanced the thermal stability and hydrophobicity of ZIF-93. The hybrid material was termed ZIF-93/11. The gas adsorption results reveal that the prepared ZIF-93/11 has an intermediate gas adsorption capacity, between those of ZIF-93 and ZIF-11. The hybrid material, ZIF-93/11, was employed as an inorganic filler in a polybenzimidazole (PBI) matrix to prepare MMMs. The resulting membrane, with a 20 wt.% loading of ZIF-93/11, exhibited improved performance in the separation of H_2_/CO_2_ at 180 °C, with a H_2_ permeability of 207 barrer and a H_2_/CO_2_ selectivity of 7.7. Figure 22 shows the comparative performance of bare PBI membranes with 20 wt.%-loaded ZIF-93, ZIF-11, and ZIF-93/11 MMMs, as well as the ZIF-93/11, which were prepared in DMAc and MeOH [180].

**Figure 23:**
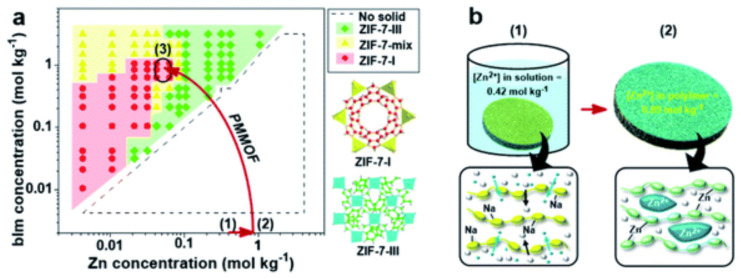
(**a**) ZIF-7 crystal-phase diagram as a function of concentrations of zinc and bIm. (**b**) Illustration of the ZIF-7 synthesis stages and the corresponding conditions during the PMMOF process. Reprinted with permission from ref. [174].

PMMOF, a polymer-modification-enabled in situ metal–organic-framework-formation method, has the potential to revolutionize the preparation of polymer/MOF mixed-matrix membranes (MMMs). However, the reaction conditions in a confined polymer void volume are different than that of synthesis in a bulk solution. In this regard, Park et. al. investigated the in situ confined formation of ZIF-7 phases in the 6FDA-DAM polymer using PMMOF. The reaction conditions were determined using a bulk-phase ZIF-7 phase diagram, and the ZIF-7 crystal phases formed during the PMMOF process were controlled (Figure 23). By a controlled synthesis, different ZIF-phases, ZIF-7-I, ZIF-7-mix, and ZIF-7-III, were synthesized. The ZIF-7-III*-based MMMs showed excellent separation performance for H_2_/CO_2_ with a selectivity of 3.8 and a permeability of 1630 barrer [174].

Boroglu et al. prepared 6FDA-DAM-ZIF-11 MMMs by varying the amount of ZIF-11 (0, 10, 20, and 30 wt.%) in the 6FDA-DAM polymer and studied their performance during hydrogen separation. The 6FDA-DAM-ZIF-11 MMMs with a 20% loading of ZIF-11 exhibited a H_2_ permeability of 272.5 barrer and a H_2_/CH_4_ selectivity of 32.8. Increasing the loading of the filler in the polymer increased the permeability to some extent, but at the same time, their selectivity barely changed [178].

**Figure 24:**
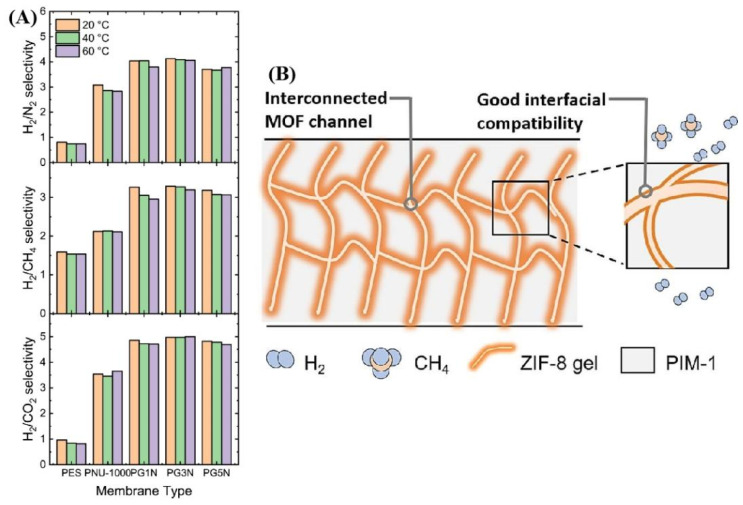
(**A**) H_2_/CO_2_, H_2_/CH_4_, and H_2_/N_2_ selectivity of MMMs (PG1N, PG3N, and PG5N) at 1 bar and 20, 40, and 60 °C. Reprinted with permission from ref. [181]. (**B**) Schematic representation of ZIF-8 gel in PIM-1 membrane matrix. Reprinted with permission from ref. [182].

Mohamed et al. fabricated a PES-based mixed-matrix membrane by the addition of graphene nanosheets (GNs) and NU-1000 (a mesoporous MOF comprising Zr). Various membranes were prepared by varying the loading of the GNs (0, 0.01, 0.03, and 0.05 wt.%) by keeping NU-1000 at 10%, abbreviated as PG1N, PG3N, and PG5N. The incorporation of GNs increased the gas permeability compared to the NU-1000 and PES membranes, owing to the barrier effect of the GNs in the matrix. Among all the prepared MMMs, the PG3N membrane displayed superior results, with a H_2_/N_2_, H_2_/CH_4_, and H_2_/CO_2_ selectivity of 4.2, 3.3, and 5, respectively, which was 23%, 40%, and 57% higher than the that of the NU-1000 membrane (Figure 24A) [181].

Sometimes, high loading of inorganic fillers causes filler aggregation due to poor interfacial compatibility between the polymer and filler. To counter this, Zhang et al. introduced the ZIF-8 gel (Figure 24B) as a novel filler to prepare MMMs with the PIM-1 polymer. The ZIF-8 gel introduces several advantages, such as interconnected MOF networks, which facilitate the formation of paths for continuous gas transport; stable and uniform dispersion; and favorable interfacial compatibility. Hence, the resulting membrane, with 31 wt.% of the ZIF-8 gel, exhibited a H_2_ permeability of 6800 barrer and a H_2_/CH_4_ selectivity of 6.78, which is significantly higher than the pure PIM-1 membrane [182]. Regmi et al. fabricated cellulose triacetate-based MMMs by introducing the combined effects of a carbon nanotube (CNT) and a titanium dioxide nanotube (TNT). The hybrid nanofiller, CNT@TNT, blended into the CTA matrix, resulted in a membrane with improved mechanical, thermal, and performance properties. The CTA-TNT@CNT improved the H_2_/CH_4_ selectivity from 36.58 (pristine CTA) to 48.43 [183].

HOFs (hydrogen-bonded organic frameworks) are novel materials with flexible and ordered porous frameworks consisting of H bonds and strong π–π interactions. Their high stability and permanent porosity make HOFs strong candidates for the large-scale development of gas purification membranes. Li wei et al. fabricated a HOF-based MMM by blending the HOF-30 filler with polyimide (PI). The resulting membrane, HOF-30@PI MMM, with a 10 wt.% loading of HOF-30, showed a high H_2_/CH_4_ selectivity of ~61.7 and permeability of 428 barrer. The novel hybrid membrane presented very high stability and consistent separation performance without any significant reduction, even after a prolonged runtime of 240 h [184]. Several reports on MMMs for H_2_/CH_4_ are summarized in Table 3. A corresponding table with additional information for the presented membranes is provided in the supporting information as Table S3.

In the present review, the fabrication and performance improvement of MMMs were briefly discussed. Inorganic fillers, such as zeolites, MOFs, silica, and carbon complexes, have been proven to enhance membrane properties in terms of permeability and selectivity and thermal, mechanical, and chemical stability. Future developments of inorganic fillers and their MMMs will facilitate the integration of MMMs into industrial operations. In a recent study, Hua et al. presented a detailed study on the progress, challenges, and future opportunities of MMMs. Despite the significant development of MMMs, scalability and defect-free manufacturing on a large scale are critical challenges that remain. This study evaluated various membrane fabrication methods, including phase inversion, solution processing, dip coating, and in situ polymerization. The fabrication of a defect-free membrane is still a challenge. They also highlighted issues associated with filler agglomeration and poor interfacial interactions between fillers and polymers. Agglomeration limits high filler loading and creates uneven membrane structures, ultimately affecting permeability and selectivity. The surface functionalization of fillers and the optimization of filler dispersion and distribution could help to mitigate these challenges. Moreover, interfacial adhesion could improve by surface modifications and in situ polymerization techniques. This study also discussed challenges in the commercialization of MMMs from the cost and engineering perspectives [193]. Consequently, there is a need for continued research with a focus on the development of novel membrane materials, improvements in fabrication technologies, and research on engineering challenges and scalability. A comparative analysis of the advantages and disadvantages of all membrane types discussed in this review, along with potential solutions, is provided in the supporting information as Table S4.

## 5. Conclusions

To overcome the challenges associated with climate change and global warming, the implementation of a hydrogen economy in place of a fossil fuel-based economy is crucial. Currently, hydrogen-production technologies mostly rely on conventional energy sources and fossil fuels. Green hydrogen production from renewable sources is a key area of development today. The utilization of hydrogen requires a high purity (~99.99%) due to applicational sensitivities. Various technologies, such as PSA and cryogenic distillation, are widely employed for large-scale processing in a number of industries. However, these require high-cost instruments and technologies, and their poor hydrogen recovery causes a ~20% loss during purification. Emerging membrane-based separation technologies are far more advantageous than conventional methods.

In this review, we discussed various potential membrane-based technologies for H_2_ purification, focusing on their preparation, materials, and separation performance. Significant research has been conducted in the area of metallic, inorganic, polymeric, and mixed-matrix membranes.

Metallic membranes provide the highest purity (~99.99%) and selectivity compared to all membrane techniques. However, the high cost of metals and metal alloys and their hydride formation, hydrogen embrittlement, and sensitivity toward CO_2_, H_2_O, H_2_S, and CO are major obstacles in their application. Palladium; metal membranes from groups IV and V and their alloy-based membranes; and amorphous metal membranes are the key research area in the field of metal membranes.

Zeolite and silica membranes are comparatively more suitable for industrial applications due to their structural tunability, high-temperature stability, and low cost. Silica membranes feature a high porosity, low material cost, and resistance to hydrogen embrittlement. However, hydrothermal instability in silica membranes is a critical drawback. On the other hand, zeolite membranes have intrinsic thermal chemical and mechanical stability. However, the complex synthesis of zeolite membranes makes them difficult to scale-up for large-scale applications. Zeolites and silica can be fabricated either in a supported or unsupported fashion. Supported membranes have better strength, selectivity, and permeability and a low cost due to their thin selective layer. Inorganic membranes are comparatively more expensive than polymeric membranes. CMSMs have high chemical and thermal resistance, a tunable pore-size distribution, and high selectivity and permeability. However, the cost of CMSMs is much higher than that of polymer membranes. The performance of CMSMs predominantly relies on the precursor material and the chemistry of the pyrolysis process. Research in CMSMs focuses on the pyrolysis chemistry, the development of precursors, hydrophobicity enhancements, and the development of composite CMSMs to tackle issues associated with water vapor.

A polymeric membrane features high flexibility, ease of processability, an application-suitable module design, ease of pre- and post-modifications, thin-film processability, good mechanical strength, good separation efficiency, and low environmental impacts, and they are more cost efficient than the inorganic membranes mentioned above. A wide range of polymeric membranes has been developed and studied for H_2_ purification and their selectivity and permeability over H_2_/CH_4_, H_2_/CO_2_, and H_2_/N_2_ separation. Polymers such as polyimide cellulose acetate, polycarbonate, polysulfone, polyetherimides, polystyrene, polyethersulfone, Matrimid, PBI, PDMS, polypropylene oxide, Hyflon^®^, Teflon^®^ AF, and PIMs are widely used for the preparation of H_2_ separation membranes. In this review, several reports were presented and summarized as per separation efficiency or selectivity over H_2_/CH_4_, H_2_/CO_2_, and H_2_/N_2_ separation. Due to their ease of processability, flexibility, and mechanical strength, polymeric membranes can be scaled-up and utilized for industrial-scale H_2_ purification. However, polymeric membranes are limited by the trade-off between permeability and selectivity, as well as issues like swelling, plasticization, thermal stability, and physical aging. There is a wide scope for research in the development of polymeric membranes with enhanced permeability, selectivity, thermal stability, and diffusivity. Additionally, the development of membrane materials that can withstand a broad range of operating conditions, particularly chemical resistance, high pressure, and temperature, and that feed streams with harsh chemicals, is necessary.

Mixed-matrix membranes (MMMs) are prepared by the incorporation of an inorganic filler into the polymer matrix to overcome the limitations of polymer and inorganic membranes and to obtain synergistic benefits from both polymers and inorganic fillers. Various MMMs were presented from the literature. Various inorganic fillers (silica, CMSs, zeolite, CNTs, and MOFs) have been employed for the fabrication of MMMs for H_2_ separation. MMMs show a higher selectivity and performance compared to their pure polymeric counterparts. However, there are some technical challenges with these, such as dispersion instability, filler agglomeration due to a high filler content, and a lack of inorganic filler–polymer interfacial adhesions. Various techniques have been developed to counter these issues, such as an in situ MOF synthesis by PMMOF (for dispersion stability) and the use of MOF gels (for interfacial compatibility and aggregation). Future research should focus on the development of new inorganic fillers and polymers to prepare MMMs that meet the commercial-scale requirements for H_2_ separation.

## Figures and Tables

**Table 1:** Gas permeability of polymers used in industrial gas separation [144,145,146].

Polymer	H_2_ (Barrer)	CO_2_ (Barrer)	N_2_ (Barrer)	CH_4_ (Barrer)
Cellulose acetate	2.63	6.3	0.21	0.21
Ethyl cellulose	87	26.5	3.2	19
Polycarbonate, brominated	NA	4.23	0.18	0.13
Polydimethylsiloxane	550	2700	250	800
Polyimide (Matrimid)	28.1	10.7	0.32	0.25
Polymethylpentene	125	84.6	6.7	14.9
Polyphenyleneoxide	113	75.8	3.81	11
Polysulfone	14	5.6	0.25	0.25
Polyetherimide	7.8	1.32	0.047	0.035
Polyethersulfone	8.96	3.38	0.129	0.112
Polystyrene (PS)	23.8	10.4	0.6	0.8
Poly (vinylidene fluoride) (Kynar)	2.4	1.2	0.7	1.3
Poly (methyl methacrylate)	2.4	0.6	1.2	0.6

**Table 2:** Gas permeability and H_2_/CH_4_ selectivity of polymers.

Polymer	Operating Conditions Temp/Pressure	Permeability P(H_2_) Barrers	Selectivity α (H_2_/CH_4_)	Year of Development	Reference
Hyflon^®^ AD60X	25 °C/1 bar	187	61.7	2007	[159]
Teflon AF-2400	25 °C/50 psig	3300	5.5	1996	[160]
Polyimide (6FDA-mMPD)	35 °C/10 atm	106	121	1992	[161]
Polyimide (6FDA-DDBT)	35 °C/10 atm	179	71	1995	[162]
Sulfonated polyimide (DAPHFDS(H))	35 °C/1 atm	52	330	2006	[163]

Note: All the presented membranes in Table 2 were prepared by the film casting method. Additional information on the polymer, membrane preparation, and operating conditions are provided in the supporting information as Table S2.

**Table 3:** Comparison of various reported mixed-matrix membranes.

Membrane/ Fabrication Technique	Polymer	Filler	Gas Pair	Selectivity	Year of Development	Ref.
NS@PBI-20	PBI	Cu MOF [Cu_2_(ndc)_2_(dabco)]_n_	H_2_/CO_2_	26.7	2015	[171]
TpPa-1(40)@PBI-BuI	PBI	COF [TpPa-1]	H_2_/CH_4_	165.5	2016	[172]
			H_2_/N_2_	79		
MMMs (20 wt.% of NUS-2@PBI)	PBI	COF [NUS-2]	H_2_/CO_2_	31.4	2016	[173]
4 wt.% UZAR-S1-PSF MMM	Psf (Udel^®^ P-3500)	UZAR-S1	H_2_/CH_4_	69.2	2011	[177]
6FDA-DAM-ZIF-11 at 20 wt.%	6FDA-DAM	ZIF-11	H_2_/CH_4_	32.8	2017	[178]
HOF-30@PI MMM	Matrimid@5218	HOF-30	H_2_/CH_4_	61.7	2022	[184]
TR-PNC	HAB-6FDA polyimide	Silica	H_2_/CO_2_	86.4	2020	[51]
			H_2_/N_2_	43.2		
Matrimid^®^ 5218/20% of DDR	Matrimid^®^ 5218	Deca-dodecasil 3R (DDR)	H_2_/CH_4_	375.27	2017	[185]
Udel^®^-Nu-6(2)	Psf	Zeolite Nu-6(2)	H_2_/CH_4_	398	2008	[186]
6FDA-Durene-ZIF-71—20%	6FDA-Durene	ZIF-71	H_2_/CH_4_	7.4	2014	[187]
(PI–6 wt.% of Cu_3_(BTC)_2_) Hollow fiber by dry/wet spinning	PI	Cu_3_(BTC)_2_	H_2_/CH_4_	240	2010	[188]
Sample M3	Psf	Mesoporous silica spheres (MSSs)	H_2_/CH_4_	79.2	2009	[189]
40 wt.% of Cu–BPY–HFS/Matrimid^®^	Matrimid^®^	Cu-BPY-HFS	H_2_/CH_4_	45.4	2008	[190]
PIM-1–g-C_3_N_4_(2.0)	PIM-1	g-C_3_N_4_ Prepared by the thermal oxidation etching method	H_2_/CH_4_	11.9	2016	[191]
50 wt.% of P5-SOF MMM	Matrimid 5218™	Pillar[5]arene (P5-SOF)	H_2_/CH_4_	600	2017	[192]

Note: All the presented membranes were prepared by film casting, followed by evaporation. Additional information on membrane preparation and operating conditions is provided in the supporting information as Table S3.

## Supplementary Materials

The following supporting information can be downloaded at: https://www.mdpi.com/article/10.3390/molecules29194676/s1, Table S1. Permeability and selectivity data of various reported metallic membranes. Table S2. Additional information for Table 2 in the main text: detailed information on membrane polymers, fabrication methods, and investigations conducted. Table S3. Additional information for Table 3 in the main text: detailed information on membrane fabrication and operating conditions during gas-separation experiments. Table S4. Advantages, disadvantages, and potential solutions for various membrane categories. References [194,195,196,197,198,199,200,201,202] are cited in the supplementary materials.
