# Supplementary material for: Hydrogen Separation Membranes: A Material Perspective
Source: Molecules. 2024 Oct 1;29(19):4676. doi: 10.3390/molecules29194676 (PMC11478078; doi:10.3390/molecules29194676)
Supplement: Supplementary file 1 [file molecules-29-04676-s001.zip › molecules-3162016-supplementary.pdf]

Supporting Information

Hydrogen Separation Membranes: A Material Perspective

Dixit V. Bhalani, and Bogyu Lim*

Department of Engineering Chemistry, Chungbuk National University (CBNU), Cheongju, Chungbuk, 28644, Republic of Korea

Corresponding author. E-mail: bglim@cbnu.ac.kr (B. Lim).

Table S1. Permeability and selectivity data of various reported metallic membranes.

**Membrane**	**Fabrication Technique**	**Thickness (µm)**	**Temperature (K) / ΔP (kPa)**	**H_2 Permeability (Barrer)**	**Selectivity H_2/N_2**	**Ref**
Pd disk	ELP	7.2	673/ 120	33,500	40,000	1
$Pd_{59}Cu_{41}$ disk	ELP	16.7	673/120	50600	105	1
Pd layer on Vycor	ELP	13	773/194	37,400	High	2
$Pd_{94}Cu_6$ on Vycor	ELP	19	673/194	11,600	High	2
$Pd_{93}Ag_7$ on Vycor	ELP	22	673/194	23000	High	2
Pd on a glass layer with pore-filled Pd	ELP	-	723/100	-	520	3
Pd on an α-Al_2O_3 alumina substrate	CVD	2	573/30	19,900	5000	4
Pd/α-Al_2O_3	CVD	1	723/68	6100	780	5
Pd–Ni alloy/α-Al_2O_3	CVD	1	723/68	6100	317	5
Pd/_x0005_ γ-Al_2O_3/α-Al_2O_3	ELP	5	780/413	18,800	600	6
$Pd_{88}Ag_{12}$/_x0005_ γ-Al_2O_3/α-Al_2O_3	ELP	11	823/413	39,700	2000	6
Pd_{65}-Cu_{35}/γ-Al_2O_3	PVD	-	673/50	-	H_2/CO_2: 6500<br>H_2/N_2: 1800	7
Pd–Ag/Hastelloy X	ELP	~4-5	673/100	-	>200,000	8
PD/PBI-HFA<br>(polymer supported)<br>Pd-$O_2$30 m (273 nm)	Vacuum electroless plating technique (VELP)	273 nm	423/800	275.5	H_2/N_2: 45.5<br>H_2/CO_2: 65<br>H_2/CO: ∞	9

Table S2. Additional information for Table 2 in the main text: detailed information on membrane polymers, fabrication methods, and investigations conducted.

Polymer	Membrane fabrication	Investigation conducted
**Hyflon® AD60X**<br><br>Hyflon® AD is a copolymer of tetrafluoroethylene and 2,4-trifluoro-5-trifluoromethoxy-1,3-dioxole, developed by Solvay.	Solution casting<br><br>[*Membrane:* G200,<br>*Solvent:* Galden,<br>*Drying:* 200°C,<br>*Thickness:* 65.2 μm]	The effect of the residual solvent on the mechanical, thermal, and transport properties of solution-cast membranes were investigated.
**Teflon AF-2400**<br><br>This an amorphous, glassy perfluorinated copolymer with 87:13 mol% of 2,2-bistrifluoromethyl-4,5-difluoro-1,3-dioxole and tetrafluoroethylene, which was developed by Du Pont.	Film casting,<br>air drying, and heating.<br><br>[*Membrane*: Dense Teflon AF-2400 film,<br>*Solvent:* perfluoro-n-methylmorpholine,<br>*Drying:* air dried at 150°C for 3 days,<br>*Thickness:* 14 μm]	Teflon AF-2400 membrane was investigated for its gas permeability, selectivity, and response to temperature and vapor activity.
**Polyimide (6FDA-mMPD)**<br><br>This was prepared from 4,4'-(hexafluoroisopropylidene)diphthali c anhydride and methyl-substituted phenylenediamines.	Film casting by doctor blade, followed by heat drying (1st: 80°C for 2 h, 2nd : water washing, 3rd : drying at 65°C for 2 h, 4th : final drying in vacuum oven at 200°C for 20 h.)	Gas permeability and solubility of prepared polyimide membranes were investigated, with key a focus on the effects of methyl substituents on the polymer backbone and its influence on permeability and selectivity.
**Polyimide (6FDA-DDBT)**<br><br>This was prepared from 2-bis(2,4-dicarboxypheny1)hexafluoropropane dianhydride (6FDA) and dimethyl-3,7-diaminodibenzothiophene-5,5-dioxide (DDBT).	Fim casting,<br>followed by drying at 473 K for 20 h in vacuum oven	The effects of large aromatic diamines on physical properties and gas permeability were investigated. The DDBT-based polyimides demonstrated superior performance for H_2/CH_4 and CO_2/CH_4 due to their nonplanar structure and high free volume.
**Sulfonated polyimide (DAPHFDS(H))**<br><br>Sulfonated homopolyimides derived from 1,4,5,8-naphthalene tetracarboxylic dianhydride (NTDA) and 2,2-bis[4-(4-aminophenoxy)phenyl] hexafluoro propane disulfonic acid (BAPHFDS).	Film casting<br>1st step: 5% solution of sulfonated polyimide (NTDA–BAPHFDS) in *m*-cresol film casted in a Petri dish (drying at 120 °C for 10 h), then soaked in methanol (at 60 °C for 1 h), followed by vacuum drying (at 150 °C for 10 h).<br>2nd step: membrane soaking in 0.1 N HCl (at room temp for 24 h), deionization by water washing, followed by vacuum drying (at 150 °C for 10 h).	The sulfonated homopolyimides were compared with non-sulfonated homopolyimides with the same backbone. The inclusion of the sulfonic acid group increased the intermolecular interaction and decreased the diffusivity of larger molecules, such as CH_4.

Note: All the references are provided in Table 2 of the main text.

Table S3. Additional information for Table 3 in the main text: detailed information on membrane fabrication and operating conditions during gas-separation experiments.

Membrane/Fabrication process	Gas-separation operating conditions Temperature/ pressure
**NS@PBI-20**<br><br>Membrane prepared by blending PBI and MOF in N-methyl-2-pyrrolidone (NMP) at 120 °C for 48 h, followed by film casting and vacuum drying (at 75 °C for 12 h with cooling up to room temperature), peeling off the film, and vacuum drying (at 200 °C for 24 h).	35°C/5 bars
**TpPa-1(40)@PBI-BuI**<br><br>The casting solution was prepared by blending a varied amount of COF in PBI-Bul from 20, 40, and 50 wt.% using the DMAc solvent, and the membranes were prepared by solution casting.	35°C/upstream pressure of 20 atm
**MMMs (20 wt % of NUS-2@PBI)**<br><br>The casting solution was prepared by dissolving PBI in NMP (at 120 °C for 48 h). Parallelly, the COF (NUS-2) was dispersed in DMF by sonication-stirring. Further, the COF dispersion was blended in the PBI solution and casted onto a glass substrate, followed by vacuum drying (at 75 °C for 12 h and later at 200 °C for 24 h).	35°C/5 bars
**4 wt.% of UZAR-S1-PSF MMM**<br><br>First, two separate solutions of the polymer and filler were prepared. A total of 0.4 g of PSF (Udel® P-3500) was dissolved in 3.6 ml of DCM (24 h of stirring). Parallelly, 4 wt% of UZAR-S1 was dispersed in DCM, at 90:10 (solvent/ filler), by sonication for 15 mins. Further, the PSF solution was blended in an UZAR-S1 solution and stirred overnight. The membrane was prepared by solution casting and drying at room temperature (overnight), followed by vacuum drying (at 120°C for overnight).	35°C/upstream pressure 2.75 bar (275 kPa)
**6FDA-DAM-ZIF-11 at 20 wt.%**<br><br>For the casting solution, two separate solutions of 6FDA-DAM (15% in NMP) and ZIF-11 in NMP were prepared. A uniform dispersion was prepared by the addition of a polymer solution in a ZIF-11 slurry with multiple cycles of sonication. The membrane was casted on a glass plate by the film applicator and dried at 70°C, followed by thermal annealing (at 150°C and 200°C).	30 °C/4 bar
**HOF-30@PI MMM**<br><br>For membrane preparation, two separate casting solutions were prepared by dispersing HOF-30 crystals in $CHCl_3$ (with stirring for 3 h and sonication for 15 min) and dissolving PI in $CHCl_3$ (with stirring for 24 h). Furthermore, the PI solution was blended into filler dispersion (with stirring for 24 h),	25°C / 1 bar

followed by film casting on a glass plate. The film was dried in vacuum oven at 100°C for 24 h and annealed.	
**TR-PNC**<br><br>The polyimide HAB-6FDA was synthesized from 3,3'-dihydroxy-4,4'-diaminobiphenyl (HAB) and 2,2-bis-(3,4-dicarboxyphenyl) hexafluoropropane dianhydride (6FDA) by chemical imidization. The filler solution (15 wt% silica/DMAc) was blended in a polymer solution and cast in a vacuum oven at 80°C. The casted films were kept at 100°C and 200°C, respectively, for 24 h in a vacuum oven. Furthermore, thermal rearrangement was carried out at 350°C in an inert atmosphere.	35°C/upstream pressure of 30 psi
**Matrimid® 5218/20% DDR**<br><br>The casting solution was prepared by dispersing 20 wt.% of DDR, with respect to Matrimid® 5218, in NMP, followed by polymer blending and film casting with a 200 μm gap. The membrane was dried at 200°C and peeled off. Moreover, the membrane was thermally treated at 250°C under a N_2 atmosphere. The final thickness of the membrane was 80–100 μm. A total of 20 wt% of DDR loading improved the permeability by up to 100% and selectivity by up to 189%.	308 K (~ 35°C)/10 bar
**Udel®-Nu-6(2)**<br><br>The zeolite crystals Nu-6(2) were dispersed in DCM (~90 wt% of the solvent/zeolite polymer) by sonication for 15 min. Then, Psf was blended and stirred at room temperature for 24 h. The membrane was prepared by film casting, followed by solvent evaporation at room temperature for 24 h. Lastly, the membrane was vacuum-dried for 24 h at 100 °C and 10 mbar.	25°C /<br>538 kPa (5.38 bar)
**6FDA-Durene-ZIF71—20%**<br><br>Two separate solutions of 6FDA-Durene and ZIF-71 were prepared in DMF. Prior to blending, the ZIF-71 solution was sonicated for 1 h and then mixed with the polymer solution. The blend was stirred for 30 mins and was film-casted. Further, the film was heated (at 80 °C for 18 h) and peeled off. Then, the film was re-dried by placing it between two aluminum foils in a vacuum oven for the complete removal of the solvent.	35°C/ 3.5 atm
**(PI–6 wt % $Cu_3(BTC)_2$)**<br><br>Poly(amic acid) was prepared from pyromellitic dianhydride (PMDA) and 4,4-oxydianiline (ODA). $Cu_3(BTC)_2$ was blended in it to prepare a Cu3(BTC)2/poly(amic acid) mixed-matrix solution. Furthermore, a hollow fiber was fabricated by dry/wet spinning, followed by temperature-programmed imidation.	298 K (25°C)/<br>1 MPa (10 bar)
**Sample M3**<br><br>The casting solution was prepared by MSS dispersion in a solvent (10/90 wt%) by sonication for 15 mins, followed by the addition of Psf (with stirring for 1 day, with 3 times the sonication for 15 min). The membrane was fabricated by film casting on a glass plate and was left for solvent evaporation (overnight). Furthermore, it was dried in a vacuum oven at a 10-mbar pressure and at 100°C (overnight). Various membranes were prepared by varying the MSS loading value (0 - 32 wt %).	35°C/<br>275 kPa (2.75 bar)

**40wt% Cu–BPY–HFS/Matrimid®**<br><br>The casting solution was prepared by dispersion of the Cu–BPY–HFS powder in chloroform by sonication (1 h), followed by the addition of Matrimid® to prepare a 10% (w/w) solution. Various membranes were prepared by varying the filler concentration (from 10 to 40 wt %). Furthermore, membranes were prepared by film casting on a glass plate and were vacuum-dried (at 50 ◦C for 3 days).	35°C/1500 Torr (around 2 bar)
**PIM-1–g-C_3N_4(2.0)**<br><br>The PIM-1 was synthesized by using equal moles of 2,3,5,6-tetra-fluoroterephthalonitrile (TFTPN, 99%) and 5,5′,6,6′-tetrahydroxy-3,3,3′,3′-tetramethyl-1,1′-<br>spirobisindane (TTSBI, 97%). For membrane preparation, two separate solutions of PIM-1 and g-C_3N_4 were prepared and blended. The solution was casted in Teflon dishes and placed for evaporation (3 days). Furthermore, the films were delaminated and vacuum-dried at 70°C.<br>The incorporation of g-C_3N_4 altered the chain packing of PIM-1, creating additional transport pathways for gas molecules. The ultramicropores in g-C_3N_4 facilitated the transport of smaller molecules, like H_2, and improved the selectivity.	30 °C/2 bar
**50 wt% P5-SOF MMM**<br><br>The casting solution was prepared by blending P5-SOF into a polymer solution (Matrimid 5218™ in chloroform). The membranes were prepared by film casting, followed by evaporation.	20°C/1 atm

Note: all the references are provided in Table 3 in the main text.

Table S4. Advantages, disadvantages, and potential solutions for various membrane categories.

**Membrane**	**Advantages**	**Disadvantages**	**Potential solutions**
**Metal Membranes**	➢ High H_2 selectivity.<br>➢ Excellent thermal stability.<br>➢ High chemical resistance.<br>➢ Suitable for high-temperature applications.	➢ High cost.<br>➢ Susceptible to poisoning. by CO and sulfur,<br>➢ Hydrogen embrittlement.	➢ Alloying: to counter the issues of hydrogen embrittlement, hydride formation, contaminant poisoning, and mechanical strength, metal alloys were prepared to improve the performance, stability, and strength and reduce the cost of the membrane.<br>➢ Fabrication of supported membranes to improve mechanical strength and reduce the costs.<br>➢ Other strategies, such as protective coating against poisoning, surface functionalization, and catalytic coating, can improve performance and durability.
**Zeolite Membranes**	➢ High chemical and chemical stability.<br>➢ Molecular sieving ability.<br>➢ Chemical resistivity.	➢ High production cost due to the requirement of defect-free membranes.<br>➢ Difficult to scale-up.	➢ Optimization of synthesis process can minimize costs and improve scalability.<br>➢ Incorporation of zeolites into the MMMs can enhance the mechanical strength and minimize manufacturing costs.
**Silica Membranes**	➢ Low cost.<br>➢ Moderate thermal stability.<br>➢ Immune to hydrogen embrittlement.	➢ Comparatively low H_2 selectivity.<br>➢ Fragility.<br>➢ Densification by moist gaseous stream (hydrothermal instability).	➢ Composite membranes can be prepared with metals and polymers to improve structural stability and reduce fragility.<br>➢ Development of hybrid silica-based membranes with well-defined pore structures could improve membrane selectivity.<br>➢ Hydrophobic modifications to reduce hydrothermal instability.
**CMSMs**	➢ High H_2 selectivity.<br>➢ High thermal stability.<br>➢ Tunable pore sizes.	➢ Complex fabrication process.<br>➢ Susceptible to pore blockage from impurities.<br>➢ Lack of reproducibility.<br>➢ Brittleness.<br>➢ High manufacturing cost.	➢ Optimization of carbonization condition and preparation of composite membranes can improve the scalability and durability of CMSMs.
**Polymeric Membranes**	➢ Ease of processing and flexibility.<br>➢ Good mechanical strength.<br>➢ Moderate selectivity.<br>➢ Ease of pre- and post-modifications.<br>➢ Thin-film processability	➢ Low thermal stability and chemical resistance.<br>➢ Swelling, plasticization, and physical aging.	➢ Membrane post-modification.<br>➢ Polymer backbone modification.<br>➢ Chemical cross-linking.<br>➢ Blending of nanomaterials.<br>➢ Fabrication of mixed-matrix membranes.

**MMMs**	➢ High selectivity and permeability compared to polymeric membranes.<br>➢ Ability to combine the processability of polymers with the high selectivity of inorganic fillers.	➢ Agglomeration of fillers.<br>➢ Poor interfacial adhesion.<br>➢ Difficulty of achieving a high filler loading.<br>➢ Limitations in scalability.	➢ Filler agglomeration can be mitigated by the surface modification of fillers.<br>➢ Filler–polymer interfacial adhesion can be improved by using compatibilizers or by using functionalized fillers.<br>➢ In situ filler growth methods and dispersion enhancements are the most promising strategies.

References

1. Gade, S.K.; Thoen, P.M.; Way, J.D. Unsupported Palladium Alloy Foil Membranes Fabricated by Electroless Plating. *J. Membr. Sci.* **2008**, *316*, 112–118.
2. Uemiya, S.; Sato, N.; Ando, H.; Kude, Y.; Matsuda, T.; Kikuchi, E. Separation of Hydrogen Through Palladium Thin Film Supported on a Porous Glass Tube. *J. Membr. Sci.* **1991**, *56*, 303–313.
3. Kuraoka, K.; Zhao, H.; Yazawa, T. Pore-Filled Palladium-Glass Composite Membranes for Hydrogen Separation by Novel Electroless Plating Technique. *J. Mater. Sci.* **2004**, *39*, 1445–1449.
4. Itoh, N.; Akiha, T.; Sato, T. Preparation of Thin Palladium Composite Membrane Tube by a CVD Technique and Its Hydrogen Permselectivity. *Catal. Today* **2005**, *104*, 231–237.
5. Jun, C.S.; Lee, K.H. Palladium and Palladium Alloy Composite Membranes Prepared by Metal-Organic Chemical Vapor Deposition Method (Cold-Wall). *J. Membr. Sci.* **2000**, *176*, 121–130.
6. Nair, B.K.R.; Choi, J.; Harold, M.P. Electroless Plating and Permeation Features of Pd and Pd/Ag Hollow Fiber Composite Membranes. *J. Membr. Sci.* **2007**, *288*, 67–84.
7. Iulianelli, A.; Ghasemzadeh, K.; Marelli, M.; Evangelisti, C. A Supported Pd-Cu/Al_2O_3 Membrane from Solvated Metal Atoms for Hydrogen Separation/Purification. *Fuel Process. Technol.* **2019**, *195*, 106141.
8. Fernandez, E.; Medrano, J.A.; Melendez, J.; Parco, M.; Viviente, J.L.; Van Sint Annaland, M.; Tanaka, D.P. Preparation and Characterization of Metallic Supported Thin Pd–Ag Membranes for Hydrogen Separation. *Chem. Eng. J.* **2016**, *305*, 182–190.
9. Kim, D.H.; Kong, S.Y.; Lee, G.H.; Yoon, C.W.; Ham, H.C.; Han, J.; Song, K.H.; Henkensmeier, D.; Choi, S.H. Effect of PBI-HFA Surface Treatments on Pd/PBI-HFA Composite Gas Separation Membranes. *Int. J. Hydrog. Energy* **2017**, *42*, 22915–22924.
